# Supplementary material for: Isolation of wheat bran-colonizing and metabolizing species from the human fecal microbiota
Source: PeerJ. 2019 Jan 25;7:e6293. doi: 10.7717/peerj.6293 (PMC6348960; doi:10.7717/peerj.6293)
Supplement: Data S2 [file peerj-07-6293-s002.pdf]

# Supplementary Information:

# Isolation of wheat bran-colonizing and metabolizing species from the human fecal microbiota

De Paepe et al., Submission to PeerJ

2018

## Availability of raw data

This Supplementary Data file contains the R code to reproduce all analyses and plots presented in the manuscript entitled "Isolation of wheat bran-colonizing and metabolizing species from the human fecal microbiota". All the required datasets are uploaded as additional supplementary files (DataS3-S9).

The raw 16S rRNA next-generation amplicon sequencing data is, moreover, deposited in the NCBI sequence read archive (SRA) under the accession number SRP091975 https://trace.ncbi.nlm.nih.gov/Traces/study/?acc=SRP091975&go=go. Raw 16S rRNA gene Sanger sequences of the isolates are supplied as a compressed folder (Sanger_isolates.zip). This folder and the raw Data files should be present in the same working directory containing this .Rmd script.

## Load the packages and datasets

The following packages are required to run the script:

```
# Formatting data
library(reshape2)
library(plyr)
library(dplyr)
library(tidyr)
library(stringi)
library(stringr)
library(splitstackshape)
library(tibble)
library(gtools)

# Plotting data
library(ggplot2)
library(gridExtra)
```

```
library(cowplot)
require(scales)
library(gplots)

# User defined functions (make sure that the R scripts below are present in t
he same working directory as this Rmarkdown script)
source('DataS11.R') # formatting of ggplot graphs
source('DataS12.R') # formatting mothur taxonomy file

# Processing of 16S rRNA gene Sanger sequencing data
library("ape")
library("phangorn", lib.loc="/usr/local/lib/R/site-library")
source("https://bioconductor.org/biocLite.R")
biocLite("DECIPHER")
biocLite("Biostrings")
biocLite("sangerseqR")
library(devtools)
install_github("Liliad/sangeranalyseR")
library(sangeranalyseR)

# Processing of next-generation 16S rRNA gene amplicon sequencing data
biocLite("phyloseq")
library("phyloseq")
library(vegan)

# Rmarkdown knitr
library(knitr)
```

Load the functional data (short chain fatty acid production in the enrichment series):

```
enrichment_SCFA <- read.csv("DataS3.csv")
```

Load the 16S rRNA gene amplicon sequencing data (read count table and taxonomy):

```
# READ COUNT DATA
shared3 <- read.csv("DataS4.csv",header=TRUE)
rownames(shared3) <- shared3[,1]
shared3 <- shared3[,-1]

# Transpose
shared3 <- as.data.frame(t(shared3))

# Apply filtering cut-offs from the waste not want not paper by McMurdie and
Holmes (2014)
shared3minsingleton <- shared3[rowSums(shared3)!=0,]
shared3minsingleton <- shared3minsingleton[rowSums(shared3minsingleton)!=1,]
minobs=1
prevalence <- apply(as.matrix(shared3minsingleton),1,function(x,minobs){sum(x
>=minobs)},minobs)/ncol(shared3minsingleton)
prevalencefilter <- prevalence>0.05
```

```r
shared3minsingletonwnwn <- shared3minsingleton[prevalencefilter,]
shared3minsingletonwnwn <- shared3minsingletonwnwn[rowSums(shared3minsingleto
nwnwn)>0.5*ncol(shared3minsingletonwnwn),]

# Transform to proportions
shared3minsingletonwnwnprop = sweep(shared3minsingletonwnwn,2,colSums(shared3
minsingletonwnwn),'/')

#TAXONOMY
RDPtax <- read.csv("DataS6.csv")
RDPtax <- splittax(RDPtax,"otu")
rownames(RDPtax) <- RDPtax$id
RDPtax <- RDPtax[,-1]
RDPtax_noprob <- dplyr::select(RDPtax,-contains("prob"))

# Formatting
RDPtax_noprob$Genus <- mapvalues(RDPtax_noprob$Genus,from=c("Enterobacteriace
ae_unclassified","Erysipelotrichaceae_unclassified","Lachnospiraceae_unclassi
fied","Lactobacillales_unclassified","Actinobacteria_unclassified","Burkholde
riales_unclassified","Clostridiales_unclassified","Ruminococcaceae_unclassifi
ed","Coriobacteriaceae_unclassified"),to=c("Enterobacteriaceae","Erysipelotri
chaceae","Lachnospiraceae","Lactobacillales","Actinobacteria","Burkholderiale
s","Clostridiales","Ruminococcaceae","Coriobacteriaceae"))

#COMBINE IN OTU TABLE
otutab3 <- dplyr::select(RDPtax_noprob,-Species)
otutab3 <- cbind(otutab3[rownames(shared3minsingletonwnwnprop),],shared3minsi
ngletonwnwnprop)

# Constructing Genus level otutable with RDP taxonomy
genusotutab3 <- aggregate(. ~ Genus, data=otutab3[,-c(1:5)], FUN=sum)
rownames(genusotutab3) <- genusotutab3[,1]
genusotutab3 <- genusotutab3[,-1]

# Constructing Family level otutable with with RDP taxonomy
familyotutab3 <- aggregate(. ~ Family, data=otutab3[,-c(1:4,6)], FUN=sum)
rownames(familyotutab3) <- familyotutab3[,1]
familyotutab3 <- familyotutab3[,-1]

# Constructing Order level otutable with with RDP taxonomy
orderotutab3 <- aggregate(. ~ Order, data=otutab3[,-c(1:3,5,6)], FUN=sum)
rownames(orderotutab3) <- orderotutab3[,1]
orderotutab3 <- orderotutab3[,-1]

# Constructing Class level otutable with with RDP taxonomy
classotutab3 <- aggregate(. ~ Class, data=otutab3[,-c(1:2,4:6)], FUN=sum)
rownames(classotutab3) <- classotutab3[,1]
classotutab3 <- classotutab3[,-1]
```

```r
# Constructing Phylum level otutable with with RDP taxonomy
phylumotutab3 <- aggregate(. ~ Phylum, data=otutab3[,-c(1,3:6)], FUN=sum)
rownames(phylumotutab3) <- phylumotutab3[,1]
phylumotutab3 <- phylumotutab3[,-1]

# ABUNDANCE DATA
shared4 <- read.csv("DataS5.csv",header=TRUE)
rownames(shared4) <- shared4[,1]
shared4 <- shared4[,-1]

# Transpose
shared4 <- as.data.frame(t(shared4))

# Apply filtering cut-offs from the waste not want not paper by McMurdie and
Holmes (2014)
shared4minsingleton <- shared4[rowSums(shared4)!=0,]
shared4minsingleton <- shared4minsingleton[rowSums(shared4minsingleton)!=1,]
minobs=1
prevalence <- apply(as.matrix(shared4minsingleton),1,function(x,minobs){sum(x
>=minobs)},minobs)/ncol(shared4minsingleton)
prevalencefilter <- prevalence>0.05
shared4minsingletonwnwn <- shared4minsingleton[prevalencefilter,]
shared4minsingletonwnwn <- shared4minsingletonwnwn[rowSums(shared4minsingleto
nwnwn)>0.5*ncol(shared4minsingletonwnwn),]

# Transform to proportions
shared4minsingletonwnwnprop = sweep(shared4minsingletonwnwn,2,colSums(shared4
minsingletonwnwn),'/')

#TAXONOMY DATA
RDPtax4 <- read.csv("DataS7.csv")
RDPtax4 <- splittax(RDPtax4,"otu")
rownames(RDPtax4) <- RDPtax4$id
RDPtax4 <- RDPtax4[,-1]
RDPtax4_noprob <- dplyr::select(RDPtax4,-contains("prob"))

RDPtax4_noprob$Genus <- mapvalues(RDPtax4_noprob$Genus,from=c("Enterobacteria
ceae_unclassified","Erysipelotrichaceae_unclassified","Lachnospiraceae_unclas
sified","Lactobacillales_unclassified","Actinobacteria_unclassified","Burkhol
deriales_unclassified","Clostridiales_unclassified","Ruminococcaceae_unclassi
fied","Coriobacteriaceae_unclassified","Lactobacillaceae_unclassified","Micro
bacteriaceae_unclassified"),to=c("Enterobacteriaceae","Erysipelotrichaceae","
Lachnospiraceae","Lactobacillales","Actinobacteria","Burkholderiales","Clostr
idiales","Ruminococcaceae","Coriobacteriaceae","Lactobacillaceae","Microbacte
riaceae"))

#COMBINE IN OTU TABLE
otutab4 <- dplyr::select(RDPtax4_noprob,-Species)
otutab4 <- cbind(otutab4[rownames(shared4minsingletonwnwnprop),],shared4minsi
ngletonwnwnprop)
```

```r
# Constructing Genus level otutable with RDP taxonomy
genusotutab4 <- aggregate(. ~ Genus, data=otutab4[,-c(1:5)], FUN=sum)
rownames(genusotutab4) <- genusotutab4[,1]
genusotutab4 <- genusotutab4[,-1]

# Constructing Family level otutable with with RDP taxonomy
familyotutab4 <- aggregate(. ~ Family, data=otutab4[,-c(1:4,6)], FUN=sum)
rownames(familyotutab4) <- familyotutab4[,1]
familyotutab4 <- familyotutab4[,-1]

# Constructing Order level otutable with with RDP taxonomy
orderotutab4 <- aggregate(. ~ Order, data=otutab4[,-c(1:3,5,6)], FUN=sum)
rownames(orderotutab4) <- orderotutab4[,1]
orderotutab4 <- orderotutab4[,-1]

# Constructing Class level otutable with with RDP taxonomy
classotutab4 <- aggregate(. ~ Class, data=otutab4[,-c(1:2,4:6)], FUN=sum)
rownames(classotutab4) <- classotutab4[,1]
classotutab4 <- classotutab4[,-1]

# Constructing Phylum level otutable with with RDP taxonomy
phylumotutab4 <- aggregate(. ~ Phylum, data=otutab4[,-c(1,3:6)], FUN=sum)
rownames(phylumotutab4) <- phylumotutab4[,1]
phylumotutab4 <- phylumotutab4[,-1]
```

Load the metadata:

```r
sampleinfo3 <- read.csv('DataS8.csv',header=TRUE)
sampleinfo3$Lumen_bran <- factor(sampleinfo3$Lumen_bran,levels=c("FS","Lumen"
,"Bran"))
sampleinfo3$pH <-factor(sampleinfo3$pH,levels=c("5.8","6.8"))

sampleinfo4 <- read.csv('DataS9.csv',header=TRUE)
sampleinfo4$Lumen_bran <- factor(sampleinfo4$Lumen_bran,levels=c("FS","Lumen"
,"Bran"))
sampleinfo4$pH <-factor(sampleinfo4$pH,levels=c("5.8","6.8"))
```

Specify color vectors:

```r
# Colors
fourcolors <- c("#DC143C","#8B5F65","#00008B","#EE9A00")
eightcolors=c("#7B4B71","#C5CD42","#C44F44","#91B7C0","#484934","#BA9C5F","#B
167C8","#69C467")

elevencolors = c("#000000","#CD6600","#36648B","#008B00","#7A378B","#838B83",
"#8B0000","#323C4D","#A03D44","#98B736","#C8ADA4")
fifteencolors <- c("#000000","#CD6600","#36648B","#008B00","#7A378B","#838B83
","#8B0000","#323C4D","#A03D44","#98B736","#C8ADA4","#BB5236","#7FCD50","#AB5
CC7","#C9A325")
```

```
thirtycolors=c("#323F24","#CB51D7","#72E245","#DE4F2D","#81DDC7","#6584C6","#CFAE3C","#914261","#BFDC86","#CDC6BE","#D3469A","#3C315A","#607B30","#DD4469","#5B9072","#CA9FC7","#7F592D","#5A656D","#D1867F","#4C2426","#79B6CE","#6E67D1","#CBDC3F","#63D98B","#97362B","#CBB37D","#7E3F85","#CF8338","#5DA73A","#CB80D0")

# Resolution of figures
dpi <- 300
```

Sanger sequences of the isolates are supplied as a compressed folder (Sanger_isolates.zip).

## SCFA production during consecutive enrichment steps

The fecal sample (enrichment step 0) and the liquid broth in the enrichment Hungate tubes after each transfer (enrichment steps 1 to 4) were sampled for SCFA analysis.

```
SCFA <- melt(enrichment_SCFA,id.vars=c("Donor","pHstart","Enrichment","pHend"))
SCFA1 <- subset(SCFA,Donor=="1")
SCFA2 <- subset(SCFA,Donor=="2")
SCFA3<- subset(SCFA,Donor=="3")
SCFA4<- subset(SCFA,Donor=="4")

p <- ggplot(data=SCFA1,aes(x=Enrichment,y=as.numeric(as.character(value))))
p <- p +geom_bar(stat="identity",aes(fill=variable))
p <- papertheme(p) +facet_grid(pHstart~.,drop=TRUE,scale="free_x",space="free_x") + scale_fill_manual(values=eightcolors,name=NULL)
p <- p+ ylab("Donor 1 SCFA (mM)") + xlab("Enrichment step") + theme(strip.text.y = element_text(angle=0),legend.justification="center") +guides(fill=guide_legend(nrow=2))
p <- p + geom_text(data=SCFA1,aes(x=Enrichment,y=100,label=pHend),size=6)
mylegendSCFA<-get_legend(p)
SCFAd1 <- p + theme(legend.position = "none",axis.title.x = element_text(color="white"))

p <- ggplot(data=SCFA1,aes(x=Enrichment,y=as.numeric(as.character(value))))
p <- p +geom_bar(stat="identity",aes(fill=variable))
p <- papertheme(p) +facet_grid(pHstart~.,drop=TRUE,scale="free_x",space="free_x") + scale_fill_manual(values=eightcolors,name=NULL)
p <- p+ ylab("Donor 1 SCFA (mM)") + xlab("Enrichment step") + theme(strip.text.y = element_text(angle=0),legend.justification="center") +guides(fill=guide_legend(nrow=2))
p <- p + geom_text(data=SCFA1,aes(x=Enrichment,y=100,label=pHend),size=6)
mylegendSCFA<-get_legend(p)
SCFAd1 <- p + theme(legend.position = "none",axis.title.x = element_text(color="white"))

p <- ggplot(data=SCFA2,aes(x=Enrichment,y=as.numeric(as.character(value))))
p <- p +geom_bar(stat="identity",aes(fill=variable))
```

```
p <- papertheme(p) +facet_grid(pHstart~.,drop=TRUE,scale="free_x",space="free
_x") + scale_fill_manual(values=eightcolors)
p <- p+ ylab("Donor 2 SCFA (mM)") + xlab("Enrichment step") + theme(legend.te
xt=element_text(face='italic'),strip.text.y = element_text(angle=0)) +guides(
fill=guide_legend(nrow=4))
p <- p + geom_text(data=SCFA2,aes(x=Enrichment,y=85,label=pHend),size=6)
SCFAd2 <- p + theme(legend.position = "none",axis.title.x = element_text(colo
r="white"))

p <- ggplot(data=SCFA3,aes(x=Enrichment,y=as.numeric(as.character(value))))
p <- p +geom_bar(stat="identity",aes(fill=variable))
p <- papertheme(p) +facet_grid(pHstart~.,drop=TRUE,scale="free_x",space="free
_x") + scale_fill_manual(values=eightcolors)
p <- p+ ylab("Donor 3 SCFA (mM)") + xlab("Enrichment step") + theme(legend.te
xt=element_text(face='italic'),strip.text.y = element_text(angle=0)) +guides(
fill=guide_legend(nrow=4))
p <- p + geom_text(data=SCFA3,aes(x=Enrichment,y=100,label=pHend),size=6)
SCFAd3 <- p + theme(legend.position = "none")

p <- ggplot(data=SCFA4,aes(x=Enrichment,y=as.numeric(as.character(value))))
p <- p +geom_bar(stat="identity",aes(fill=variable))
p <- papertheme(p) +facet_grid(pHstart~.,drop=TRUE,scale="free_x",space="free
_x") + scale_fill_manual(values=eightcolors)
p <- p+ ylab("Donor 4 SCFA (mM)") + xlab("Enrichment step") + theme(legend.te
xt=element_text(face='italic'),strip.text.y = element_text(angle=0)) +guides(
fill=guide_legend(nrow=4))
p <- p + geom_text(data=SCFA4,aes(x=Enrichment,y=100,label=pHend),size=6)
SCFAd4 <- p + theme(legend.position = "none")

tiff("Figure2.tiff",width=10*dpi,height=16*dpi,res=dpi,compression="lzw")
cowplot::plot_grid(plot_grid(SCFAd1,SCFAd2,SCFAd3,SCFAd4,nrow=2,ncol=2,rel_he
ights =c(0.5,0.5,0.5,0.5),rel_widths=c(0.5,0.5,0.5,0.5),labels='AUTO',label_s
ize=20,label_fontfamily='sans'),mylegendSCFA,nrow=2,rel_heights=c(0.95,0.05))
dev.off()

## png
##   2

knitr::include_graphics("Figure2.tiff")
```

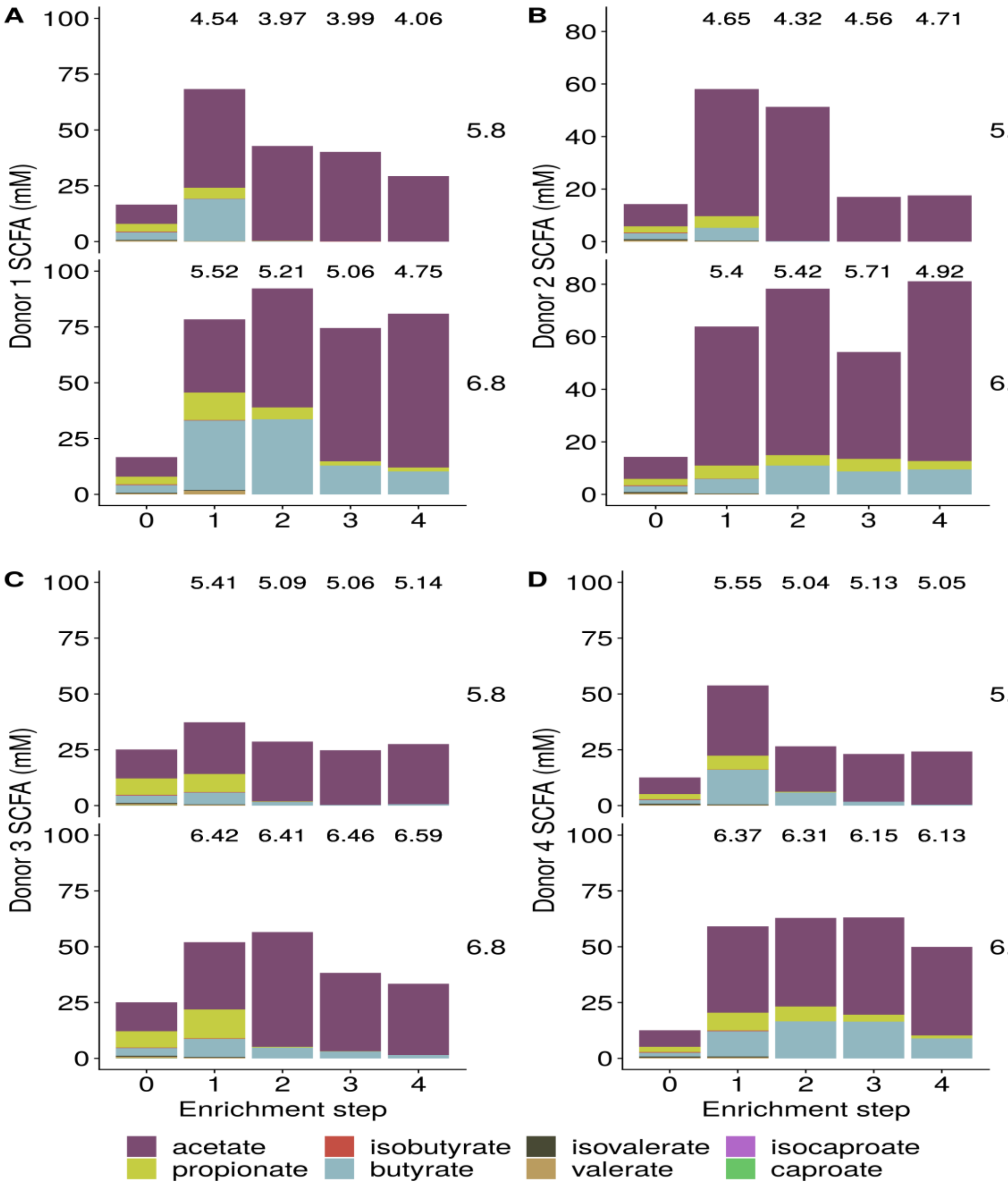


***Figure 2: Short Chain Fatty Acid( (SCFA) production by the fecal microbiota derived from four different donors during enrichment with wheat bran as the sole nutrient source.*** *The fecal sample (enrichment step 0) was incubated with wheat bran for 24 h (enrichment step 1), after which the wheat bran residue was washed to remove loosely attached bacteria and used to seed a new incubation (enrichment step 2). This procedure was repeated two more times (enrichment step 3 and 4). The pH is indicated on top of the stacked bars and decreased considerably comparing to the starting pH (5.8 and 6.8).*

## Richness and Diversity indices calculated on the next-generation 16S rRNA gene amplicon sequencing confirm the ‘enrichment’

The outcome of the enrichment procedure was assessed by computing richness (Chao1 Richness estimator) and diversity (Shannon, Simpson, inverse Simpson and Fisher alpha) estimators using vegan_2.4-4 (Oksanen et al. 2016).

```
dataunnormalised <- as.data.frame(t(shared3))
# Calculate the chao index
chaoest=apply(dataunnormalised,1,function(x) estimateR(x))[2,]
# Annotate the dataframe
richnessdf <- cbind(chaoest,sampleinfo3)
richnessdf$Enrichment <-as.numeric(as.character(richnessdf$Enrichment))
richness1 <- subset(richnessdf,Donor=="1"&Nameshared!="Donor2_low4_Brep.V4")
richness2 <- subset(richnessdf,Donor=="2")
richness3<- subset(richnessdf,Donor=="3")
# Repeat for donor 4
dataunnormalised <- as.data.frame(t(shared4))
chaoest=apply(dataunnormalised,1,function(x) estimateR(x))[2,]
richnessdf4 <- cbind(chaoest,sampleinfo4)
richnessdf4$Enrichment <- as.numeric(as.character(richnessdf4$Enrichment))
richness4 <- subset(richnessdf4,Donor=="4")

# Evolution of the chao richness during consecutive enrichment steps in donor
1 (Figure 3A in the manuscript)
p <- ggplot(data=richness1,aes(x=Enrichment,y=as.numeric(as.character(chaoest
))))
p <- p +geom_line(aes(group=Lumen_bran,linetype=Lumen_bran))+geom_point(aes(s
hape=Lumen_bran),size=5)
p <- papertheme(p) +facet_grid(pH~.,drop=TRUE,scale="free_x",space="free_x")
+ scale_fill_manual(values=eightcolors,name=NULL) + scale_shape_manual(name=N
ULL,values=c(15,16,17))+ scale_linetype_manual(name=NULL,values=c(0,1,2))
p <- p+ ylab("Chao1 richness estimator \n Donor1") + xlab("Enrichment step")
+ theme(strip.text.y = element_text(angle=0)) +guides(fill=guide_legend(nrow=
2))+ theme(legend.key.width = unit(2,"cm"))
richplot1 <- p + theme(legend.position = "none",axis.title.x = element_text(c
olor="white"))

# Evolution of the chao richness during consecutive enrichment steps in three
donors
richness <- rbind(richness1,richness2,richness3)
richness$Donor <- as.factor(richness$Donor)

p <- ggplot(data=richness,aes(x=Enrichment,y=as.numeric(as.character(chaoest)
)))
p <- p +geom_line(aes(group=interaction(Lumen_bran,Donor),color=Donor))+geom_
point(aes(shape=Lumen_bran,color=Donor),size=5)
p <- papertheme(p) +facet_grid(pH~.,drop=TRUE,scale="free_x",space="free_x")
+ scale_color_manual(values=fourcolors,name=NULL)
```

```r
p <- p+ ylab("Chao richness estimator") + xlab("Enrichment step") + theme(leg
end.text=element_text(face='italic'),strip.text.y = element_text(angle=0)) +g
uides(fill=guide_legend(nrow=2))
richplot <- p + theme(legend.position = "none",axis.title.x = element_text(co
lor="white"))

dataunnormalised <- as.data.frame(t(shared3))
# Calculate different diversity indices
divdf <- data.frame(shannon=apply(dataunnormalised,1,function(x) vegan::diver
sity(x)),simpson=apply(dataunnormalised,1,function(x) vegan::diversity(x,"sim
p")),invsimpson=apply(dataunnormalised,1,function(x) vegan::diversity(x,"inv"
)),fisher=apply(dataunnormalised,1,function(x) vegan::fisher.alpha(x)))
# Annotate the dataframe
divdf <- cbind(divdf,sampleinfo3)
divdf$Enrichment <- as.numeric(as.character(divdf$Enrichment))
divdf1 <- subset(divdf,Donor=="1"&Nameshared!="Donor2_low4_Brep.V4")
divdf1$Lumen_bran <- mapvalues(divdf1$Lumen_bran,from="FS",to="Fecal sample")

divdf2 <- subset(divdf,Donor=="2")
divdf3<- subset(divdf,Donor=="3")
# Repeat for donor 4
dataunnormalised <- as.data.frame(t(shared4))
divdf4 <- data.frame(shannon=apply(dataunnormalised,1,function(x) vegan::dive
rsity(x)),simpson=apply(dataunnormalised,1,function(x) vegan::diversity(x,"si
mp")),invsimpson=apply(dataunnormalised,1,function(x) vegan::diversity(x,"inv
")),fisher=apply(dataunnormalised,1,function(x) vegan::fisher.alpha(x)))
divdf4 <- cbind(divdf4,sampleinfo4)

# Evolution of the diversity indices during consecutive enrichment steps in d
onor 1 (Figure 3B in the manuscript)
p <- ggplot(data=divdf1,aes(x=Enrichment,y=as.numeric(as.character(shannon)))
)
p <- p +geom_line(aes(group=Lumen_bran,linetype=Lumen_bran))+geom_point(aes(s
hape=Lumen_bran),size=5)
p <- papertheme(p) +facet_grid(pH~.,drop=TRUE,scale="free_x",space="free_x")
+ scale_fill_manual(values=eightcolors,name=NULL) + scale_shape_manual(name=N
ULL,values=c(15,16,17)) + scale_linetype_manual(name=NULL,values=c(0,1,2))
p <- p+ ylab("Shannon diversity index \n Donor 1") + xlab("Enrichment step")
+ theme(strip.text.y = element_text(angle=0)) +guides(fill=guide_legend(nrow=
2)) + theme(legend.justification="center") + theme(legend.key.width = unit(2,
"cm"))
mylegendrichndiv <- get_legend(p)
divplot1 <- p + theme(legend.position = "none")
# Evolution of the diversity indices during consecutive enrichment steps thre
e donors
divdf <- rbind(divdf1,divdf2,divdf3)
divdf$Donor <- as.factor(divdf$Donor)

p <- ggplot(data=divdf,aes(x=Enrichment,y=as.numeric(as.character(shannon))))
p <- p +geom_line(aes(group=interaction(Lumen_bran,Donor),color=Donor))+geom_
```

```
point(aes(shape=Lumen_bran,color=Donor),size=5)
p <- papertheme(p) +facet_grid(pH~.,drop=TRUE,scale="free_x",space="free_x")
+ scale_color_manual(values=fourcolors,name=NULL)
p <- p+ ylab("Chao richness estimator") + xlab("Enrichment step") + theme(leg
end.text=element_text(face='italic'),strip.text.y = element_text(angle=0)) +g
uides(fill=guide_legend(nrow=2))
p <- p + theme(legend.position = "none",axis.title.x = element_text(color="wh
ite"))

tiff("Figure3.tiff",width=6*dpi,height=14*dpi,res=dpi,compression="lzw")
cowplot::plot_grid(plot_grid(richplot1,divplot1,ncol=1,rel_heights = c(0.5,0.
5),labels='AUTO',label_size=20,label_fontfamily='sans'),mylegendrichndiv,nrow
=2,rel_heights=c(0.95,0.05))
dev.off()

## png
##   2

include_graphics("Figure3.tiff")
```

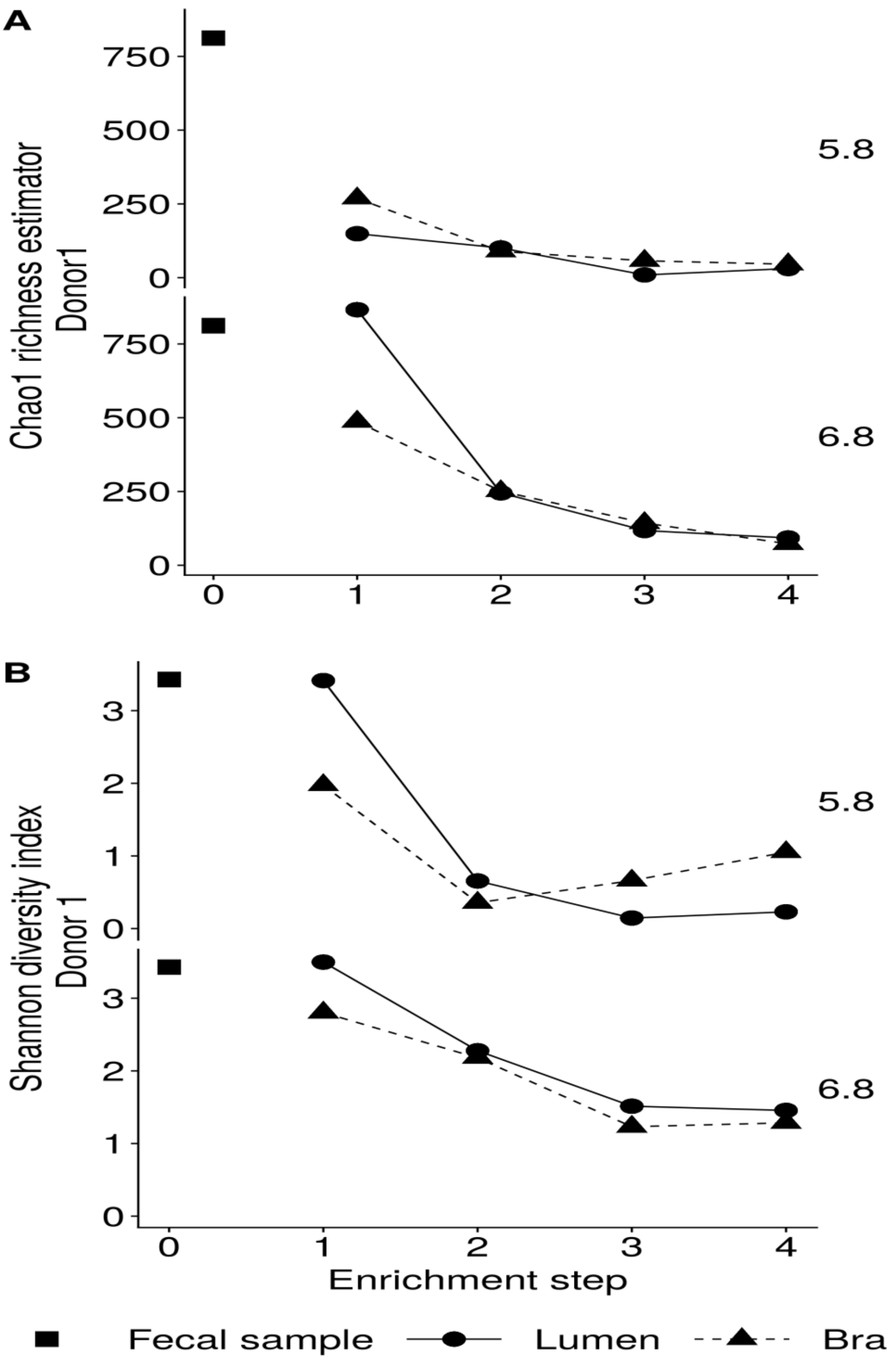


***Figure 3: Reduction in the microbial community richness (Chao 1 richness estimator) and diversity (Shannon diversity index) during consecutive enrichment steps with wheat bran as the sole nutrient source, as shown for donor 1.*** *The fecal sample (enrichment step 0) was incubated with wheat bran for 24 h (enrichment step 1), after which the wheat bran residue was washed to remove loosely attached bacteria and used to seed a new incubation (enrichment step 2). This procedure was repeated two more times (enrichment step 3 and 4).*

## Identification of the enriched species by next-generation 16S rRNA gene amplicon sequencing

Bar graphs of the proporional microbial community composition at phylum, genus, and species level were created to demonstrate community shifts during consecutive enrichment steps with wheat bran as the sole nutrient source.

```
otuspec <- phylumotutab3
otuspec <- 100*otuspec
otuspec <- rbind(names=colnames(otuspec),otuspec)
otuspec <- mutate(as.data.frame(t(otuspec)),donor=sampleinfo3$Donor,LBM=sampl
einfo3$Lumen_bran,enrichment=sampleinfo3$Enrichment,pH=sampleinfo3$pH)
otuspec <- melt(otuspec,id.vars=c('donor','LBM','enrichment','pH','names'))
otuspec1 <- subset(otuspec,donor=="1"&enrichment!="c"&names!="Donor2_low4_Bre
p.V4")
otuspec2 <- subset(otuspec,donor=="2"&enrichment!="c"&names!="Donor2_low4_Bre
p.V4")
otuspec3<- subset(otuspec,donor=="3"&enrichment!="c"&names!="Donor2_low4_Brep
.V4")

# Donor1
p <- ggplot(data=otuspec1,aes(x=LBM,y=as.numeric(as.character(value))))
p <- p +geom_bar(stat="identity",aes(fill=variable))
p <- papertheme(p) +facet_grid(pH~enrichment,drop=TRUE,scale="free_x",space="
free_x") + scale_fill_manual(values=elevencolors)
p <- p+ ylab("Relative abundance (%)") + xlab(NULL) + theme(legend.text=eleme
nt_text(face='italic'),strip.text.y = element_text(angle=0)) +guides(fill=gui
de_legend(nrow=4))

# Donor2
p <- ggplot(data=otuspec2,aes(x=LBM,y=as.numeric(as.character(value))))
p <- p +geom_bar(stat="identity",aes(fill=variable))
p <- papertheme(p) +facet_grid(pH~enrichment,drop=TRUE,scale="free_x",space="
free_x") + scale_fill_manual(values=elevencolors)
p <- p+ ylab("Relative abundance (%)") + xlab(NULL) + theme(legend.text=eleme
nt_text(face='italic'),strip.text.y = element_text(angle=0)) +guides(fill=gui
de_legend(nrow=4))

#Donor3
p <- ggplot(data=otuspec3,aes(x=LBM,y=as.numeric(as.character(value))))
p <- p +geom_bar(stat="identity",aes(fill=variable))
p <- papertheme(p) +facet_grid(pH~enrichment,drop=TRUE,scale="free_x",space="
free_x") + scale_fill_manual(values=elevencolors)
p <- p+ ylab("Relative abundance (%)") + xlab(NULL) + theme(legend.text=eleme
nt_text(face='italic'),strip.text.y = element_text(angle=0)) +guides(fill=gui
de_legend(nrow=4))

otuspec<- genusotutab3
otuspec <- cbind(sampleinfo3,t(otuspec))
```

```
otuspec <- subset(otuspec,Enrichment!="c")
otuspec <- otuspec[,-c(1:length(colnames(sampleinfo3)))]
otuspec <- data.frame(t(otuspec))
selecttop20par <- rowSums(otuspec)
otuspec <- tibble::rownames_to_column(otuspec)
otuspec <- as.data.frame(otuspec  %>% top_n(13,selecttop20par))
other <- 1-colSums(otuspec[,-1])
rownames(otuspec) <- otuspec$rowname
otuspec <- otuspec[,-1]
otuspec <- rbind(otuspec,other)
rownames(otuspec)[nrow(otuspec)] <- "Other"
otuspec <- 100*otuspec

sampleinfosub3 <- subset(sampleinfo3,Enrichment!="c")
otuspec <- mutate(as.data.frame(t(otuspec)),donor=sampleinfosub3$Donor,LBM=sa
mpleinfosub3$Lumen_bran,enrichment=sampleinfosub3$Enrichment,pH=sampleinfosub
3$pH,names=colnames(otuspec))
otuspec <- melt(otuspec,id.vars=c('donor','LBM','enrichment','pH','names'))
otuspec1 <- subset(otuspec,donor=="1"&names!="Donor2_low4_Brep.V4")
otuspec2 <- subset(otuspec,donor=="2"&names!="Donor2_low4_Brep.V4")
otuspec3<- subset(otuspec,donor=="3"&names!="Donor2_low4_Brep.V4")

# Donor1
p <- ggplot(data=otuspec1,aes(x=LBM,y=as.numeric(as.character(value))))
p <- p +geom_bar(stat="identity",aes(fill=variable))
p <- papertheme(p) +facet_grid(pH~enrichment,drop=TRUE,scale="free_x",space="
free_x") + scale_fill_manual(values=fifteencolors,name="Genus")
p <- p+ ylab("Relative abundance (%)") + xlab(NULL) + theme(legend.text=eleme
nt_text(face='italic'),strip.text.y = element_text(angle=0),legend.position="
right") +guides(fill=guide_legend(ncol=1))
bargraph1genus <- p

# Donor2
p <- ggplot(data=otuspec2,aes(x=LBM,y=as.numeric(as.character(value))))
p <- p +geom_bar(stat="identity",aes(fill=variable))
p <- papertheme(p) +facet_grid(pH~enrichment,drop=TRUE,scale="free_x",space="
free_x") + scale_fill_manual(values=fifteencolors,name="Genus")
p <- p+ ylab("Relative abundance (%)") + xlab(NULL) + theme(legend.text=eleme
nt_text(face='italic'),strip.text.y = element_text(angle=0),legend.position="
right") +guides(fill=guide_legend(ncol=1))

# Donor3
p <- ggplot(data=otuspec3,aes(x=LBM,y=as.numeric(as.character(value))))
p <- p +geom_bar(stat="identity",aes(fill=variable))
p <- papertheme(p) +facet_grid(pH~enrichment,drop=TRUE,scale="free_x",space="
free_x") + scale_fill_manual(values=fifteencolors,name="Genus")
p <- p+ ylab("Relative abundance (%)") + xlab(NULL) + theme(legend.text=eleme
nt_text(face='italic'),strip.text.y = element_text(angle=0),legend.position="
right") +guides(fill=guide_legend(ncol=1))
```

```
# Donor4
otuspec<- genusotutab4
selecttop20par <- rowSums(otuspec)
otuspec <- tibble::rownames_to_column(otuspec)
otuspec <- as.data.frame(otuspec  %>% top_n(13,selecttop20par))
other <- 1-colSums(otuspec[,-1])
rownames(otuspec) <- otuspec$rowname
otuspec <- otuspec[,-1]
otuspec <- rbind(otuspec,other)
rownames(otuspec)[nrow(otuspec)] <- "Other"
otuspec <- otuspec*100

otuspec <- mutate(as.data.frame(t(otuspec)),donor=sampleinfo4$Donor,LBM=sampl
einfo4$Lumen_bran,enrichment=sampleinfo4$Enrichment,pH=sampleinfo4$pH,names=c
olnames(otuspec))
otuspec4 <- melt(otuspec,id.vars=c('donor','LBM','enrichment','pH','names'))

p <- ggplot(data=otuspec4,aes(x=LBM,y=as.numeric(as.character(value))))
p <- p +geom_bar(stat="identity",aes(fill=variable))
p <- papertheme(p) +facet_grid(pH~enrichment,drop=TRUE,scale="free_x",space="
free_x") + scale_fill_manual(values=fifteencolors,name="Genus")
p <- p+ ylab("Relative abundance (%)") + xlab(NULL) + theme(legend.text=eleme
nt_text(face='italic'),strip.text.y = element_text(angle=0),legend.position="
right") +guides(fill=guide_legend(ncol=1))

# Species level annotation of OTUs (verified by BLAST and RDP)
label3donorsillu <- c("OTU1 Bifidobacterium sp.","OTU2 Enterobacteriaceae sp.
","OTU3 Escherichia/Shigella sp.","OTU4 Lactobacillus sp.","OTU5 Faecalibacte
rium prausnitzii","OTU6 Prevotella copri","OTU7 Faecalibacterium prausnitzii"
,"OTU8 Pediococcus sp.","OTU9 E. rectale/R. faecis","OTU10 Fusobacterium mort
iferum","OTU11 Bacteroides dorei","OTU12 Dialister sp.","OTU13 Sutterella wad
sworthensis","OTU14 Lactobacillus sp.","OTU15","OTU16 Bacteroides eggerthii",
"OTU17 Blautia wexlerae","OTU18 Lactobacillus fermentus","OTU19 Fusicatenibac
ter saccharivorans","OTU20 Roseburia faecis","Other")
otuspec<-otutab3[,-c(1:6)]
otuspec <- cbind(sampleinfo3,t(otuspec))
otuspec <- subset(otuspec,Enrichment!="c")
otuspec <- otuspec[,-c(1:length(colnames(sampleinfo3)))]
otuspec <- data.frame(t(otuspec))
selecttop20par <- rowSums(otuspec)
otuspec <- tibble::rownames_to_column(otuspec)
otuspec <- as.data.frame(otuspec  %>% top_n(20,selecttop20par))
other <- 1-colSums(otuspec[,-1])
rownames(otuspec) <- otuspec$rowname
otuspec <- otuspec[,-1]
otuspec <- rbind(otuspec,other)
rownames(otuspec)[nrow(otuspec)] <- "Other"
otuspec <- 100*otuspec

sampleinfosub3 <- subset(sampleinfo3,Enrichment!="c")
```

```r
otuspec <- mutate(as.data.frame(t(otuspec)),donor=sampleinfosub3$Donor,LBM=sa
mpleinfosub3$Lumen_bran,enrichment=sampleinfosub3$Enrichment,pH=sampleinfosub
3$pH,names=colnames(otuspec))
otuspec <- melt(otuspec,id.vars=c('donor','LBM','enrichment','pH','names'))
otuspec1 <- subset(otuspec,donor=="1"&names!="Donor2_low4_Brep.V4")
otuspec2 <- subset(otuspec,donor=="2"&names!="Donor2_low4_Brep.V4")
otuspec3<- subset(otuspec,donor=="3"&names!="Donor2_low4_Brep.V4")

# Donor1
p <- ggplot(data=otuspec1,aes(x=LBM,y=as.numeric(as.character(value))))
p <- p +geom_bar(stat="identity",aes(fill=variable))
p <- papertheme(p) +facet_grid(pH~enrichment,drop=TRUE,scale="free_x",space="
free_x") + scale_fill_manual(values=thirtycolors,name="Species",labels=label3
donorsillu)
p <- p+ ylab("Relative abundance (%)") + xlab(NULL) + theme(legend.text=eleme
nt_text(face='italic'),strip.text.y = element_text(angle=0),legend.position="
right") +guides(fill=guide_legend(ncol=1))
bargraph1species <- p

#Donor2
p <- ggplot(data=otuspec2,aes(x=LBM,y=as.numeric(as.character(value))))
p <- p +geom_bar(stat="identity",aes(fill=variable))
p <- papertheme(p) +facet_grid(pH~enrichment,drop=TRUE,scale="free_x",space="
free_x") + scale_fill_manual(values=thirtycolors,name="Species")
p <- p+ ylab("Relative abundance (%)") + xlab(NULL) + theme(legend.text=eleme
nt_text(face='italic'),legend.position="right",strip.text.y = element_text(an
gle=0)) +guides(fill=guide_legend(ncol=1))

# Donor3
p <- ggplot(data=otuspec3,aes(x=LBM,y=as.numeric(as.character(value))))
p <- p +geom_bar(stat="identity",aes(fill=variable))
p <- papertheme(p) +facet_grid(pH~enrichment,drop=TRUE,scale="free_x",space="
free_x") + scale_fill_manual(values=thirtycolors,name="Species")
p <- p+ ylab("Relative abundance (%)") + xlab(NULL) + theme(legend.text=eleme
nt_text(face='italic'),strip.text.y = element_text(angle=0),legend.position="
right") +guides(fill=guide_legend(ncol=1))

tiff("Figure4.tiff",width=16*dpi,height=14*dpi,res=dpi,compression="lzw")
cowplot::plot_grid(bargraph1genus,bargraph1species,nrow=2,rel_heights =c(0.5,
0.5),labels='AUTO',label_size=20,label_fontfamily='sans',align="v",axis="1")
dev.off()

## png
##   2

knitr::include_graphics("Figure4.tiff")
```

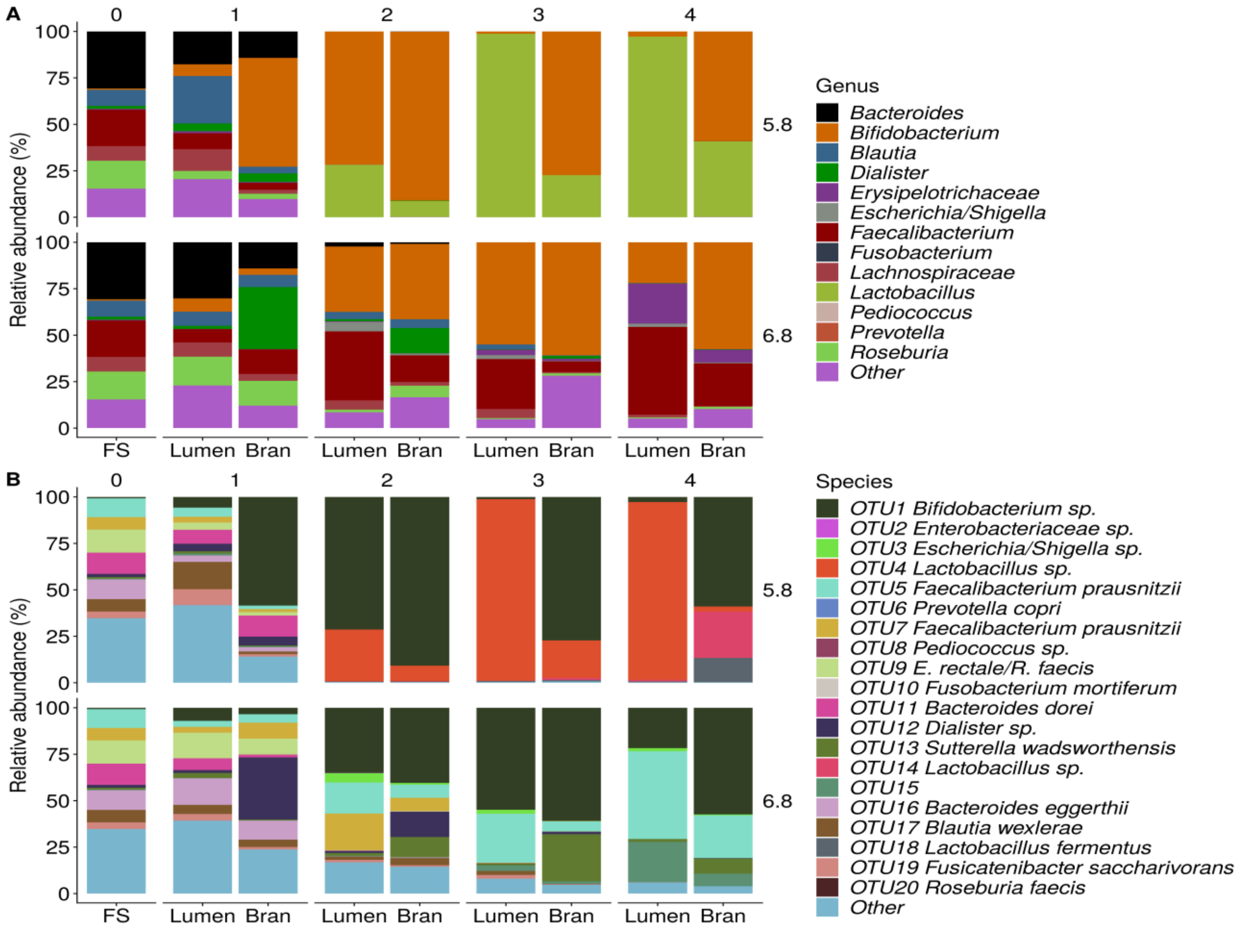


***Figure 4: Shifts in genus (A) and species (B) level microbial community composition of donor 1 during consecutive enrichment steps with wheat bran as the sole nutrient source.*** *The fecal sample (FS; enrichment step 0) was incubated with wheat bran for 24 h (enrichment step 1), after which the wheat bran residue was washed to remove loosely attached bacteria and used to seed a new incubation (enrichment step 2). This procedure was repeated two more times (enrichment step 3 and 4). Family level taxa appearing in the genus level plots should be interpreted as unclassified genus belonging to the respective family.*

```
# Genus level
otuspec<- genusotutab3
otuspec <- cbind(sampleinfo3,t(otuspec))
otuspec <- subset(otuspec,Enrichment=="4"|Enrichment=="0")
otuspec <- otuspec[,-c(1:length(colnames(sampleinfo3)))]
otuspec <- data.frame(t(otuspec))
selecttop20par <- rowSums(otuspec)
otuspec <- tibble::rownames_to_column(otuspec)
otuspec <- as.data.frame(otuspec  %>% top_n(13,selecttop20par))
other <- 1-colSums(otuspec[,-1])
rownames(otuspec) <- otuspec$rowname
otuspec <- otuspec[,-1]
```

```r
otuspec <- rbind(otuspec,other)
rownames(otuspec)[nrow(otuspec)] <- "Other"
otuspec <- 100*otuspec

sampleinfosub3 <- subset(sampleinfo3,Enrichment=="4"|Enrichment=="0")
otuspec <- mutate(as.data.frame(t(otuspec)),donor=sampleinfosub3$Donor,LBM=sa
mpleinfosub3$Lumen_bran,enrichment=sampleinfosub3$Enrichment,pH=sampleinfosub
3$pH,names=colnames(otuspec))
otuspec <- melt(otuspec,id.vars=c('donor','LBM','enrichment','pH','names'))
otuspec23 <- subset(otuspec,donor=="2"|donor=="3")
otuspec23$donor <- mapvalues(otuspec23$donor,from=c("2","3"),to=c("Donor2","D
onor3"))

p <- ggplot(data=otuspec23,aes(x=LBM,y=as.numeric(as.character(value))))
p <- p +geom_bar(stat="identity",aes(fill=variable))
p <- papertheme(p) +facet_grid(pH~donor,drop=TRUE,scale="free_x",space="free_
x") + scale_fill_manual(values=fifteencolors,name="Genus")
p <- p+ ylab("Relative abundance (%)") + xlab(NULL) + theme(legend.text=eleme
nt_text(face='italic'),strip.text.y = element_text(angle=0),legend.position="
right") +guides(fill=guide_legend(ncol=1))
bargraph23genus <- p

# Species level
otuspec<- otutab3[,-c(1:6)]
otuspec <- cbind(sampleinfo3,t(otuspec))
otuspec <- subset(otuspec,Enrichment=="4"|Enrichment=="0")
otuspec <- otuspec[,-c(1:length(colnames(sampleinfo3)))]
otuspec <- data.frame(t(otuspec))
selecttop20par <- rowSums(otuspec)
otuspec <- tibble::rownames_to_column(otuspec)
otuspec <- as.data.frame(otuspec  %>% top_n(20,selecttop20par))
other <- 1-colSums(otuspec[,-1])
rownames(otuspec) <- otuspec$rowname
otuspec <- otuspec[,-1]
otuspec <- rbind(otuspec,other)
rownames(otuspec)[nrow(otuspec)] <- "Other"
otuspec <- 100*otuspec

sampleinfosub3 <- subset(sampleinfo3,Enrichment=="4"|Enrichment=="0")
otuspec <- mutate(as.data.frame(t(otuspec)),donor=sampleinfosub3$Donor,LBM=sa
mpleinfosub3$Lumen_bran,enrichment=sampleinfosub3$Enrichment,pH=sampleinfosub
3$pH,names=colnames(otuspec))
otuspec <- melt(otuspec,id.vars=c('donor','LBM','enrichment','pH','names'))
otuspec23 <- subset(otuspec,donor=="2"|donor=="3")
otuspec23$donor <- mapvalues(otuspec23$donor,from=c("2","3"),to=c("Donor2","D
onor3"))

p <- ggplot(data=otuspec23,aes(x=LBM,y=as.numeric(as.character(value))))
p <- p +geom_bar(stat="identity",aes(fill=variable))
p <- papertheme(p) +facet_grid(pH~donor,drop=TRUE,scale="free_x",space="free_
```

```
x") + scale_fill_manual(values=thirtycolors,name="Species",labels=label3donor
sillu)
p <- p+ ylab("Relative abundance (%)") + xlab(NULL) + theme(legend.text=eleme
nt_text(face='italic'),strip.text.y = element_text(angle=0),legend.position="
right") +guides(fill=guide_legend(ncol=1))
bargraph23species <- p

tiff("Figure5.tiff",width=12*dpi,height=16*dpi,res=dpi,compression="lzw")
cowplot::plot_grid(bargraph23genus,bargraph23species,nrow=2,rel_heights =c(0.
5,0.5),labels='AUTO',label_size=20,label_fontfamily='sans',align="v",axis="1"
)
dev.off()

## png
##   2

include_graphics("Figure5.tiff")
```

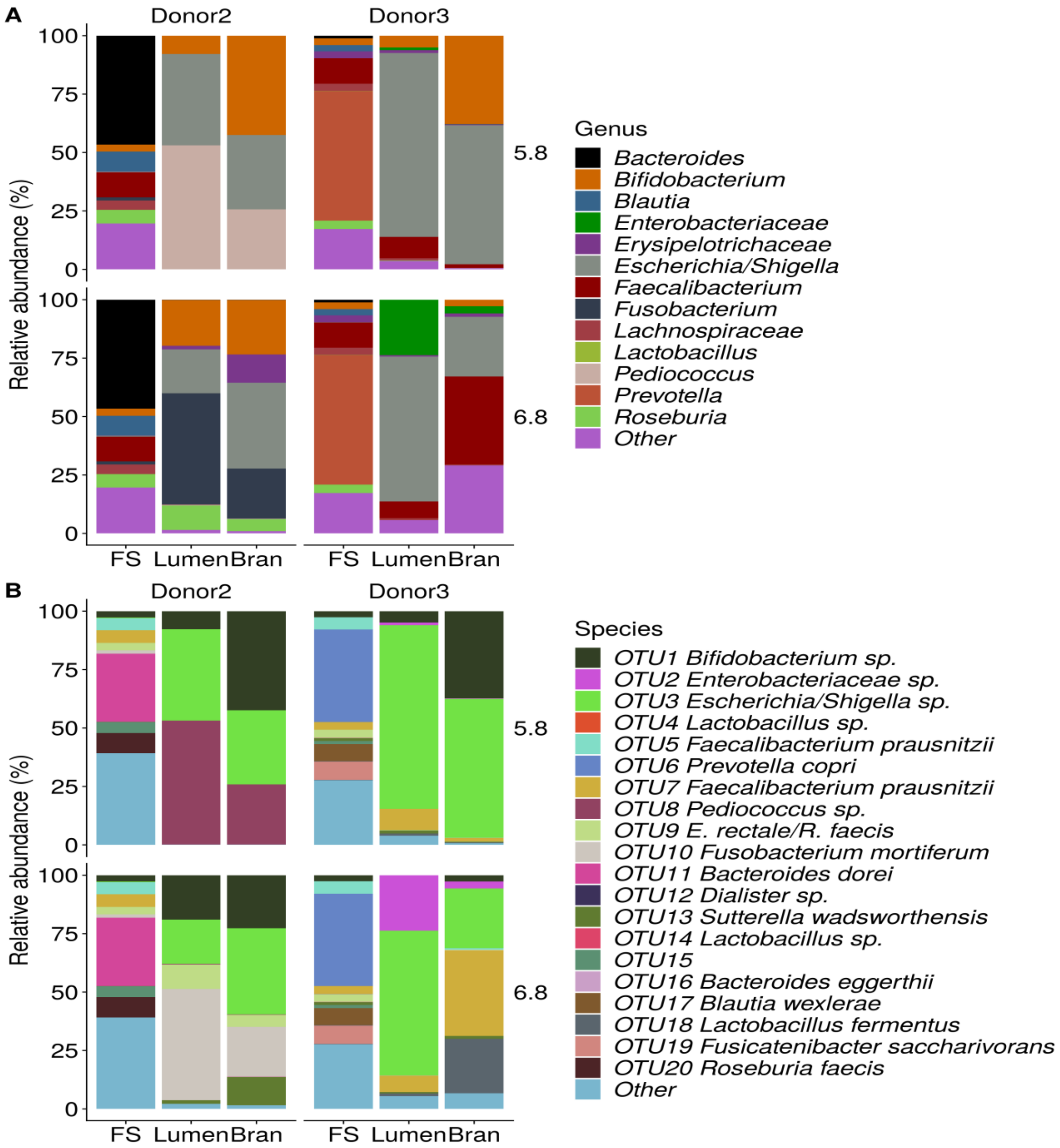


***Figure 5: Shifts in genus (A) and species (B) level microbial community composition of donor 2 and 3 after four enrichment steps with wheat bran as the sole nutrient source.*** *The fecal sample (FS; enrichment step 0) was incubated with wheat bran for 24 h (enrichment step 1), after which the wheat bran residue was washed to remove loosely attached bacteria and used to seed a new incubation (enrichment step 2). This procedure was repeated two more times (enrichment step 3 and 4). Only the final enrichment step and FS are shown in this plot. Family level taxa appearing in the genus level plots should be interpreted as unclassified genus belonging to the respective family.*

```r
# Define additional colors
fifteencolorsbis <- c("#C9A325","#000000","#CD6600","#36648B","#008B00","#838B83","#8B0000","#323C4D","#A03D44","#98B736","#7FCD50","#BB5236","#C8ADA4","#AB5CC7","#7A378B")

# Species level annotation of OTUs (verified by BLAST and RDP)
label4donorsillu <- c("OTU1 Escherichia/Shigella sp.","OTU2 Bifidobacterium sp.","OTU3 Faecalibacterium prausnitzii","OTU4 Lactobacillus gasseri","OTU5 Clostridium XVII","OTU6 Lachnospiraceae sp.","OTU7 Fusicatenibacter saccharivorans","OTU8 Clostridium XIVa sp.","OTU9 Collinsella aerofaciens","OTU10 Eubacterium rectale","OTU11 Bacteroides vulgatus","OTU12 Bifidobacterium longum","OTU13 Gemmiger formicilis","OTU14 Dialister invisus","Other")

otuspec<- genusotutab4
selecttop20par <- rowSums(otuspec)
otuspec <- tibble::rownames_to_column(otuspec)
otuspec <- as.data.frame(otuspec  %>% top_n(13,selecttop20par))
other <- 1-colSums(otuspec[,-1])
rownames(otuspec) <- otuspec$rowname
otuspec <- otuspec[,-1]
otuspec <- rbind(otuspec,other)
rownames(otuspec)[nrow(otuspec)] <- "Other"
otuspec <- otuspec*100

otuspec <- mutate(as.data.frame(t(otuspec)),donor=sampleinfo4$Donor,LBM=sampleinfo4$Lumen_bran,enrichment=sampleinfo4$Enrichment,pH=sampleinfo4$pH,names=colnames(otuspec))
otuspec <- otuspec[c(5,6,9,10),]
otuspec4 <- melt(otuspec,id.vars=c('donor','LBM','enrichment','pH','names'))

p <- ggplot(data=otuspec4,aes(x=LBM,y=as.numeric(as.character(value))))
p <- p +geom_bar(stat="identity",aes(fill=variable))
p <- papertheme(p) +facet_grid(pH~.,drop=TRUE,scale="free_x",space="free_x")
+ scale_fill_manual(values=fifteencolorsbis,name="Genus")
p <- p+ ylab("Relative abundance (%)") + xlab(NULL) + theme(legend.text=element_text(face='italic'),legend.position="right",strip.text.y = element_text(angle=0)) +guides(fill=guide_legend(ncol=1))
bargraph4genus <-p

# Species
otuspec<- otutab4[,-c(1:6)]
selecttop20par <- rowSums(otuspec)
otuspec <- tibble::rownames_to_column(otuspec)
otuspec <- as.data.frame(otuspec  %>% top_n(14,selecttop20par))
other <- 1-colSums(otuspec[,-1])
rownames(otuspec) <- otuspec$rowname
otuspec <- otuspec[,-1]
otuspec <- rbind(otuspec,other)
rownames(otuspec)[nrow(otuspec)] <- "Other"
otuspec <- otuspec*100
```

```
otuspec <- mutate(as.data.frame(t(otuspec)),donor=sampleinfo4$Donor,LBM=sampl
einfo4$Lumen_bran,enrichment=sampleinfo4$Enrichment,pH=sampleinfo4$pH,names=c
olnames(otuspec))
otuspec <- otuspec[c(5,6,9,10),]
otuspec4 <- melt(otuspec,id.vars=c('donor','LBM','enrichment','pH','names'))

p <- ggplot(data=otuspec4,aes(x=LBM,y=as.numeric(as.character(value))))
p <- p +geom_bar(stat="identity",aes(fill=variable))
p <- papertheme(p) +facet_grid(pH~.,drop=TRUE,scale="free_x",space="free_x")
+ scale_fill_manual(values=thirtycolors,name="Species",label=label4donorsillu
)
p <- p+ ylab("Relative abundance (%)") + xlab(NULL) + theme(legend.text=eleme
nt_text(face='italic'),legend.position="right",strip.text.y = element_text(an
gle=0)) +guides(fill=guide_legend(ncol=1))
bargraph4species <- p

tiff("Figure6.tiff",width=12*dpi,height=10*dpi,res=dpi,compression="lzw")
cowplot::plot_grid(bargraph4genus,bargraph4species,nrow=2,rel_heights =c(0.5,
0.5),labels='AUTO',label_size=20,label_fontfamily='sans',align="v",axis="1")
dev.off()

## png
##   2

include_graphics("Figure6.tiff")
```

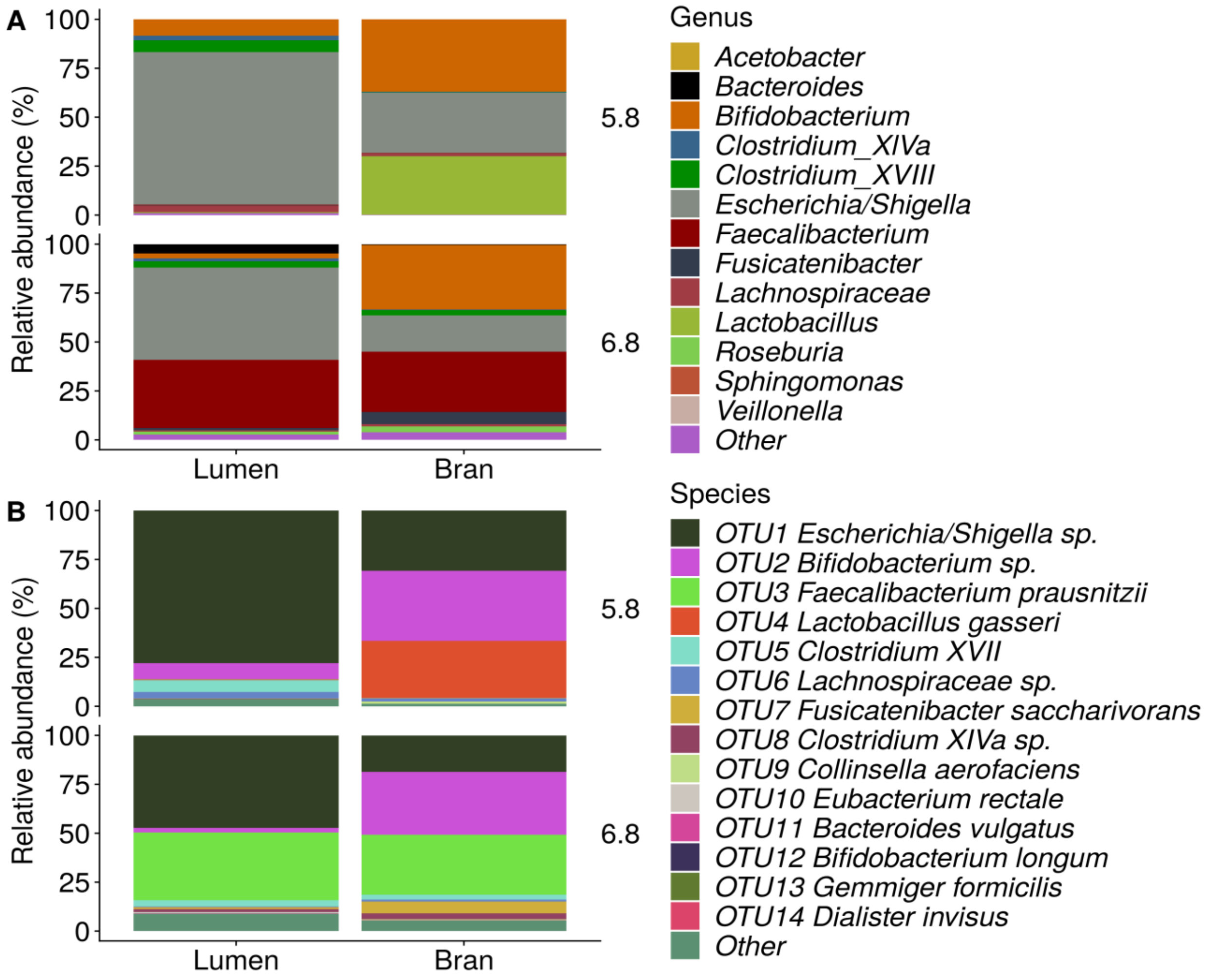


***Figure 6: Genus (A) and species (B) level microbial community composition of donor 4 after the final enrichment step with wheat bran as the sole nutrient source.*** *The fecal sample (FS; enrichment step 0) was incubated with wheat bran for 24 h (enrichment step 1), after which the wheat bran residue was washed to remove loosely attached bacteria and used to seed a new incubation (enrichment step 2). This procedure was repeated two more times (enrichment step 3 and 4). Only the final enrichment step was shown in this plot, due to a low read number (<100 reads) in the fecal sample. Family level taxa appearing in the genus level plots should be interpreted as unclassified genus belonging to the respective family.*

## Build consensus sanger sequences

The OTUs, resulting from 16S rRNA gene amplicon sequencing of the consecutive enrichments were compared to the 16S rRNA gene Sanger reads of the obtained isolates by means of a phylogenetic placement analysis. Forward and reverse Sanger sequences for each donor were grouped into separate files and reverse complements and summary statistics were obtained using the mothur software package (v.1.39.5) (Schloss et al. 2009). The 515F-806R primer pair, used for Illumina MiSeq 16S rRNA gene amplicon sequencing was located

in the Sanger reads (forward and reverse compliment). In case both primers were not present on one and the same read (either forward, or reverse), consensus sequences (contigs) were generated using the sangeranalyseR package (version 0.1.0) (Lanfear 2015). Contigs with more than 100 degenerated positions, indicative of a poor quality alignment, were omitted.

```
# Summarize data
sf = summarise.abi.folder("Sanger_isolates")
```

```
## [1] "Looking for .ab1 files..."
## [1] "Found 256 .ab1 files..."
## [1] "Loading reads..."
## [1] "Calculating read summaries..."
## [1] "Cleaning up"
```

```
sf$summaries
```

```
##                       file.path     folder.name  file.name raw.length
## 1   Sanger_isolates/100F.F.ab1 Sanger_isolates 100F.F.ab1        891
## 2   Sanger_isolates/100R.R.ab1 Sanger_isolates 100R.R.ab1        485
## 3   Sanger_isolates/101F.F.ab1 Sanger_isolates 101F.F.ab1        793
## 4   Sanger_isolates/101R.R.ab1 Sanger_isolates 101R.R.ab1       1128
## 5   Sanger_isolates/102F.F.ab1 Sanger_isolates 102F.F.ab1       1164
## 6   Sanger_isolates/102R.R.ab1 Sanger_isolates 102R.R.ab1       1100
## 7   Sanger_isolates/103F.F.ab1 Sanger_isolates 103F.F.ab1       1117
## 8   Sanger_isolates/103R.R.ab1 Sanger_isolates 103R.R.ab1       1115
## 9   Sanger_isolates/104F.F.ab1 Sanger_isolates 104F.F.ab1       1165
## 10  Sanger_isolates/104R.R.ab1 Sanger_isolates 104R.R.ab1       1166
## 11  Sanger_isolates/105F.F.ab1 Sanger_isolates 105F.F.ab1       1074
## 12  Sanger_isolates/105R.R.ab1 Sanger_isolates 105R.R.ab1        805
## 13  Sanger_isolates/106F.F.ab1 Sanger_isolates 106F.F.ab1        894
## 14  Sanger_isolates/106R.R.ab1 Sanger_isolates 106R.R.ab1       1116
## 15  Sanger_isolates/107F.F.ab1 Sanger_isolates 107F.F.ab1       1021
## 16  Sanger_isolates/107R.R.ab1 Sanger_isolates 107R.R.ab1       1133
## 17  Sanger_isolates/108F.F.ab1 Sanger_isolates 108F.F.ab1        962
## 18  Sanger_isolates/108R.R.ab1 Sanger_isolates 108R.R.ab1       1088
## 19  Sanger_isolates/109F.F.ab1 Sanger_isolates 109F.F.ab1       1126
## 20  Sanger_isolates/109R.R.ab1 Sanger_isolates 109R.R.ab1       1148
## 21   Sanger_isolates/10F.F.ab1 Sanger_isolates  10F.F.ab1       1045
## 22   Sanger_isolates/10R.R.ab1 Sanger_isolates  10R.R.ab1       1104
## 23  Sanger_isolates/110F.F.ab1 Sanger_isolates 110F.F.ab1        876
## 24  Sanger_isolates/110R.R.ab1 Sanger_isolates 110R.R.ab1       1033
## 25   Sanger_isolates/11F.F.ab1 Sanger_isolates  11F.F.ab1        546
## 26   Sanger_isolates/11R.R.ab1 Sanger_isolates  11R.R.ab1        683
## 27  Sanger_isolates/128F.F.ab1 Sanger_isolates 128F.F.ab1       1017
## 28  Sanger_isolates/128R.R.ab1 Sanger_isolates 128R.R.ab1       1196
## 29  Sanger_isolates/129F.F.ab1 Sanger_isolates 129F.F.ab1        711
## 30  Sanger_isolates/129R.R.ab1 Sanger_isolates 129R.R.ab1       1073
## 31  Sanger_isolates/130F.F.ab1 Sanger_isolates 130F.F.ab1        546
## 32  Sanger_isolates/130R.R.ab1 Sanger_isolates 130R.R.ab1        489
## 33  Sanger_isolates/131F.F.ab1 Sanger_isolates 131F.F.ab1        977
```

```
## 34  Sanger_isolates/131R.R.ab1 Sanger_isolates 131R.R.ab1       1105
## 35  Sanger_isolates/132F.F.ab1 Sanger_isolates 132F.F.ab1        200
## 36  Sanger_isolates/132R.R.ab1 Sanger_isolates 132R.R.ab1        199
## 37  Sanger_isolates/133F.F.ab1 Sanger_isolates 133F.F.ab1        199
## 38  Sanger_isolates/133R.R.ab1 Sanger_isolates 133R.R.ab1       1001
## 39  Sanger_isolates/134F.F.ab1 Sanger_isolates 134F.F.ab1        287
## 40  Sanger_isolates/134R.R.ab1 Sanger_isolates 134R.R.ab1        341
## 41  Sanger_isolates/135F.F.ab1 Sanger_isolates 135F.F.ab1        993
## 42  Sanger_isolates/135R.R.ab1 Sanger_isolates 135R.R.ab1        129
## 43  Sanger_isolates/136F.F.ab1 Sanger_isolates 136F.F.ab1        920
## 44  Sanger_isolates/136R.R.ab1 Sanger_isolates 136R.R.ab1       1109
## 45  Sanger_isolates/137F.F.ab1 Sanger_isolates 137F.F.ab1        735
## 46  Sanger_isolates/137R.R.ab1 Sanger_isolates 137R.R.ab1        691
## 47  Sanger_isolates/138F.F.ab1 Sanger_isolates 138F.F.ab1        714
## 48  Sanger_isolates/138R.R.ab1 Sanger_isolates 138R.R.ab1        872
## 49  Sanger_isolates/139F.F.ab1 Sanger_isolates 139F.F.ab1        610
## 50  Sanger_isolates/139R.R.ab1 Sanger_isolates 139R.R.ab1        382
## 51   Sanger_isolates/13F.F.ab1 Sanger_isolates  13F.F.ab1        604
## 52   Sanger_isolates/13R.R.ab1 Sanger_isolates  13R.R.ab1        957
## 53  Sanger_isolates/140F.F.ab1 Sanger_isolates 140F.F.ab1        542
## 54  Sanger_isolates/140R.R.ab1 Sanger_isolates 140R.R.ab1        943
## 55  Sanger_isolates/141F.F.ab1 Sanger_isolates 141F.F.ab1       1060
## 56  Sanger_isolates/141R.R.ab1 Sanger_isolates 141R.R.ab1        779
## 57  Sanger_isolates/142F.F.ab1 Sanger_isolates 142F.F.ab1        680
## 58  Sanger_isolates/142R.R.ab1 Sanger_isolates 142R.R.ab1        707
## 59  Sanger_isolates/143F.F.ab1 Sanger_isolates 143F.F.ab1       1067
## 60  Sanger_isolates/143R.R.ab1 Sanger_isolates 143R.R.ab1       1019
## 61  Sanger_isolates/144F.F.ab1 Sanger_isolates 144F.F.ab1        905
## 62  Sanger_isolates/144R.R.ab1 Sanger_isolates 144R.R.ab1       1059
## 63  Sanger_isolates/145F.F.ab1 Sanger_isolates 145F.F.ab1       1049
## 64  Sanger_isolates/145R.R.ab1 Sanger_isolates 145R.R.ab1        650
## 65  Sanger_isolates/146F.F.ab1 Sanger_isolates 146F.F.ab1       1121
## 66  Sanger_isolates/146R.R.ab1 Sanger_isolates 146R.R.ab1       1145
## 67  Sanger_isolates/147F.F.ab1 Sanger_isolates 147F.F.ab1       1002
## 68  Sanger_isolates/147R.R.ab1 Sanger_isolates 147R.R.ab1       1050
## 69  Sanger_isolates/148F.F.ab1 Sanger_isolates 148F.F.ab1        736
## 70  Sanger_isolates/148R.R.ab1 Sanger_isolates 148R.R.ab1        390
## 71  Sanger_isolates/149F.F.ab1 Sanger_isolates 149F.F.ab1        660
## 72  Sanger_isolates/149R.R.ab1 Sanger_isolates 149R.R.ab1       1139
## 73  Sanger_isolates/150F.F.ab1 Sanger_isolates 150F.F.ab1        505
## 74  Sanger_isolates/150R.R.ab1 Sanger_isolates 150R.R.ab1        937
## 75  Sanger_isolates/151F.F.ab1 Sanger_isolates 151F.F.ab1        706
## 76  Sanger_isolates/151R.R.ab1 Sanger_isolates 151R.R.ab1        999
## 77  Sanger_isolates/152F.F.ab1 Sanger_isolates 152F.F.ab1        614
## 78  Sanger_isolates/152R.R.ab1 Sanger_isolates 152R.R.ab1        843
## 79  Sanger_isolates/153F.F.ab1 Sanger_isolates 153F.F.ab1        703
## 80  Sanger_isolates/153R.R.ab1 Sanger_isolates 153R.R.ab1        933
## 81  Sanger_isolates/154F.F.ab1 Sanger_isolates 154F.F.ab1        943
## 82  Sanger_isolates/154R.R.ab1 Sanger_isolates 154R.R.ab1       1221
## 83  Sanger_isolates/155F.F.ab1 Sanger_isolates 155F.F.ab1        752
```

```
## 84  Sanger_isolates/155R.R.ab1 Sanger_isolates 155R.R.ab1       1122
## 85  Sanger_isolates/156F.F.ab1 Sanger_isolates 156F.F.ab1        610
## 86  Sanger_isolates/156R.R.ab1 Sanger_isolates 156R.R.ab1       1034
## 87  Sanger_isolates/157F.F.ab1 Sanger_isolates 157F.F.ab1        712
## 88  Sanger_isolates/157R.R.ab1 Sanger_isolates 157R.R.ab1       1115
## 89  Sanger_isolates/158F.F.ab1 Sanger_isolates 158F.F.ab1        874
## 90  Sanger_isolates/158R.R.ab1 Sanger_isolates 158R.R.ab1        990
## 91  Sanger_isolates/159F.F.ab1 Sanger_isolates 159F.F.ab1        702
## 92  Sanger_isolates/159R.R.ab1 Sanger_isolates 159R.R.ab1       1103
## 93   Sanger_isolates/15F.F.ab1 Sanger_isolates  15F.F.ab1        829
## 94   Sanger_isolates/15R.R.ab1 Sanger_isolates  15R.R.ab1        923
## 95  Sanger_isolates/160F.F.ab1 Sanger_isolates 160F.F.ab1        778
## 96  Sanger_isolates/160R.R.ab1 Sanger_isolates 160R.R.ab1       1157
## 97  Sanger_isolates/161F.F.ab1 Sanger_isolates 161F.F.ab1        796
## 98  Sanger_isolates/161R.R.ab1 Sanger_isolates 161R.R.ab1       1015
## 99  Sanger_isolates/162F.F.ab1 Sanger_isolates 162F.F.ab1        665
## 100 Sanger_isolates/162R.R.ab1 Sanger_isolates 162R.R.ab1        581
## 101 Sanger_isolates/163F.F.ab1 Sanger_isolates 163F.F.ab1        806
## 102 Sanger_isolates/163R.R.ab1 Sanger_isolates 163R.R.ab1        837
## 103 Sanger_isolates/181F.F.ab1 Sanger_isolates 181F.F.ab1       1149
## 104 Sanger_isolates/181R.R.ab1 Sanger_isolates 181R.R.ab1       1188
## 105 Sanger_isolates/182F.F.ab1 Sanger_isolates 182F.F.ab1       1050
## 106 Sanger_isolates/182R.R.ab1 Sanger_isolates 182R.R.ab1        815
## 107 Sanger_isolates/183F.F.ab1 Sanger_isolates 183F.F.ab1       1044
## 108 Sanger_isolates/183R.R.ab1 Sanger_isolates 183R.R.ab1       1190
## 109 Sanger_isolates/184F.F.ab1 Sanger_isolates 184F.F.ab1        916
## 110 Sanger_isolates/184R.R.ab1 Sanger_isolates 184R.R.ab1       1151
## 111 Sanger_isolates/185F.F.ab1 Sanger_isolates 185F.F.ab1        757
## 112 Sanger_isolates/185R.R.ab1 Sanger_isolates 185R.R.ab1       1147
## 113 Sanger_isolates/186F.F.ab1 Sanger_isolates 186F.F.ab1        595
## 114 Sanger_isolates/186R.R.ab1 Sanger_isolates 186R.R.ab1       1023
## 115 Sanger_isolates/187F.F.ab1 Sanger_isolates 187F.F.ab1       1142
## 116 Sanger_isolates/187R.R.ab1 Sanger_isolates 187R.R.ab1       1131
## 117 Sanger_isolates/188F.F.ab1 Sanger_isolates 188F.F.ab1       1095
## 118 Sanger_isolates/188R.R.ab1 Sanger_isolates 188R.R.ab1       1023
## 119 Sanger_isolates/189F.F.ab1 Sanger_isolates 189F.F.ab1       1049
## 120 Sanger_isolates/189R.R.ab1 Sanger_isolates 189R.R.ab1       1029
## 121  Sanger_isolates/18F.F.ab1 Sanger_isolates  18F.F.ab1        655
## 122  Sanger_isolates/18R.R.ab1 Sanger_isolates  18R.R.ab1        999
## 123 Sanger_isolates/190F.F.ab1 Sanger_isolates 190F.F.ab1       1063
## 124 Sanger_isolates/190R.R.ab1 Sanger_isolates 190R.R.ab1        709
## 125 Sanger_isolates/191F.F.ab1 Sanger_isolates 191F.F.ab1       1021
## 126 Sanger_isolates/191R.R.ab1 Sanger_isolates 191R.R.ab1       1153
## 127 Sanger_isolates/192F.F.ab1 Sanger_isolates 192F.F.ab1       1208
## 128 Sanger_isolates/192R.R.ab1 Sanger_isolates 192R.R.ab1       1157
## 129 Sanger_isolates/193F.F.ab1 Sanger_isolates 193F.F.ab1       1009
## 130 Sanger_isolates/193R.R.ab1 Sanger_isolates 193R.R.ab1       1121
## 131 Sanger_isolates/194F.F.ab1 Sanger_isolates 194F.F.ab1       1020
## 132 Sanger_isolates/194R.R.ab1 Sanger_isolates 194R.R.ab1       1137
## 133 Sanger_isolates/195F.F.ab1 Sanger_isolates 195F.F.ab1       1091
```

```
## 134 Sanger_isolates/195R.R.ab1 Sanger_isolates 195R.R.ab1       1107
## 135 Sanger_isolates/196F.F.ab1 Sanger_isolates 196F.F.ab1       1175
## 136 Sanger_isolates/196R.R.ab1 Sanger_isolates 196R.R.ab1       1095
## 137 Sanger_isolates/197F.F.ab1 Sanger_isolates 197F.F.ab1        676
## 138 Sanger_isolates/197R.R.ab1 Sanger_isolates 197R.R.ab1       1179
## 139 Sanger_isolates/198F.F.ab1 Sanger_isolates 198F.F.ab1       1100
## 140 Sanger_isolates/198R.R.ab1 Sanger_isolates 198R.R.ab1        928
## 141 Sanger_isolates/199F.F.ab1 Sanger_isolates 199F.F.ab1        609
## 142 Sanger_isolates/199R.R.ab1 Sanger_isolates 199R.R.ab1        906
## 143  Sanger_isolates/19F.F.ab1 Sanger_isolates  19F.F.ab1       1060
## 144  Sanger_isolates/19R.R.ab1 Sanger_isolates  19R.R.ab1       1160
## 145 Sanger_isolates/200F.F.ab1 Sanger_isolates 200F.F.ab1       1217
## 146 Sanger_isolates/200R.R.ab1 Sanger_isolates 200R.R.ab1        368
## 147 Sanger_isolates/201F.F.ab1 Sanger_isolates 201F.F.ab1       1183
## 148 Sanger_isolates/201R.R.ab1 Sanger_isolates 201R.R.ab1        965
## 149 Sanger_isolates/202F.F.ab1 Sanger_isolates 202F.F.ab1       1227
## 150 Sanger_isolates/202R.R.ab1 Sanger_isolates 202R.R.ab1       1117
## 151 Sanger_isolates/203F.F.ab1 Sanger_isolates 203F.F.ab1        482
## 152 Sanger_isolates/203R.R.ab1 Sanger_isolates 203R.R.ab1        987
## 153 Sanger_isolates/204F.F.ab1 Sanger_isolates 204F.F.ab1        603
## 154 Sanger_isolates/204R.R.ab1 Sanger_isolates 204R.R.ab1        584
## 155 Sanger_isolates/205F.F.ab1 Sanger_isolates 205F.F.ab1        487
## 156 Sanger_isolates/205R.R.ab1 Sanger_isolates 205R.R.ab1        566
## 157 Sanger_isolates/206F.F.ab1 Sanger_isolates 206F.F.ab1       1183
## 158 Sanger_isolates/206R.R.ab1 Sanger_isolates 206R.R.ab1       1190
## 159  Sanger_isolates/21F.F.ab1 Sanger_isolates  21F.F.ab1        903
## 160  Sanger_isolates/21R.R.ab1 Sanger_isolates  21R.R.ab1        918
## 161  Sanger_isolates/25F.F.ab1 Sanger_isolates  25F.F.ab1        904
## 162  Sanger_isolates/25R.R.ab1 Sanger_isolates  25R.R.ab1        931
## 163  Sanger_isolates/26F.F.ab1 Sanger_isolates  26F.F.ab1       1127
## 164  Sanger_isolates/26R.R.ab1 Sanger_isolates  26R.R.ab1        813
## 165  Sanger_isolates/27F.F.ab1 Sanger_isolates  27F.F.ab1        966
## 166  Sanger_isolates/27R.R.ab1 Sanger_isolates  27R.R.ab1        824
## 167  Sanger_isolates/28F.F.ab1 Sanger_isolates  28F.F.ab1        908
## 168  Sanger_isolates/28R.R.ab1 Sanger_isolates  28R.R.ab1       1017
## 169  Sanger_isolates/29F.F.ab1 Sanger_isolates  29F.F.ab1        966
## 170  Sanger_isolates/29R.R.ab1 Sanger_isolates  29R.R.ab1       1109
## 171  Sanger_isolates/30F.F.ab1 Sanger_isolates  30F.F.ab1        914
## 172  Sanger_isolates/30R.R.ab1 Sanger_isolates  30R.R.ab1        945
## 173  Sanger_isolates/31F.F.ab1 Sanger_isolates  31F.F.ab1        970
## 174  Sanger_isolates/31R.R.ab1 Sanger_isolates  31R.R.ab1        999
## 175  Sanger_isolates/32F.F.ab1 Sanger_isolates  32F.F.ab1       1045
## 176  Sanger_isolates/32R.R.ab1 Sanger_isolates  32R.R.ab1       1234
## 177  Sanger_isolates/33F.F.ab1 Sanger_isolates  33F.F.ab1        996
## 178  Sanger_isolates/33R.R.ab1 Sanger_isolates  33R.R.ab1        825
## 179  Sanger_isolates/34F.F.ab1 Sanger_isolates  34F.F.ab1        933
## 180  Sanger_isolates/34R.R.ab1 Sanger_isolates  34R.R.ab1       1143
## 181  Sanger_isolates/35F.F.ab1 Sanger_isolates  35F.F.ab1        920
## 182  Sanger_isolates/35R.R.ab1 Sanger_isolates  35R.R.ab1       1161
## 183  Sanger_isolates/36F.F.ab1 Sanger_isolates  36F.F.ab1        843
```

```
## 184  Sanger_isolates/36R.R.ab1 Sanger_isolates  36R.R.ab1       1091
## 185  Sanger_isolates/37F.F.ab1 Sanger_isolates  37F.F.ab1        942
## 186  Sanger_isolates/37R.R.ab1 Sanger_isolates  37R.R.ab1       1018
## 187  Sanger_isolates/38F.F.ab1 Sanger_isolates  38F.F.ab1        771
## 188  Sanger_isolates/38R.R.ab1 Sanger_isolates  38R.R.ab1        610
## 189  Sanger_isolates/39F.F.ab1 Sanger_isolates  39F.F.ab1        481
## 190  Sanger_isolates/39R.R.ab1 Sanger_isolates  39R.R.ab1        460
## 191  Sanger_isolates/40F.F.ab1 Sanger_isolates  40F.F.ab1       1039
## 192  Sanger_isolates/40R.R.ab1 Sanger_isolates  40R.R.ab1       1046
## 193  Sanger_isolates/41F.F.ab1 Sanger_isolates  41F.F.ab1       1172
## 194  Sanger_isolates/41R.R.ab1 Sanger_isolates  41R.R.ab1       1114
## 195  Sanger_isolates/42F.F.ab1 Sanger_isolates  42F.F.ab1        988
## 196  Sanger_isolates/42R.R.ab1 Sanger_isolates  42R.R.ab1        980
## 197  Sanger_isolates/43F.F.ab1 Sanger_isolates  43F.F.ab1       1119
## 198  Sanger_isolates/43R.R.ab1 Sanger_isolates  43R.R.ab1       1164
## 199  Sanger_isolates/44F.F.ab1 Sanger_isolates  44F.F.ab1        956
## 200  Sanger_isolates/44R.R.ab1 Sanger_isolates  44R.R.ab1       1067
## 201  Sanger_isolates/45F.F.ab1 Sanger_isolates  45F.F.ab1       1088
## 202  Sanger_isolates/45R.R.ab1 Sanger_isolates  45R.R.ab1        878
## 203  Sanger_isolates/46F.F.ab1 Sanger_isolates  46F.F.ab1        990
## 204  Sanger_isolates/46R.R.ab1 Sanger_isolates  46R.R.ab1       1086
## 205  Sanger_isolates/47F.F.ab1 Sanger_isolates  47F.F.ab1        752
## 206  Sanger_isolates/47R.R.ab1 Sanger_isolates  47R.R.ab1        582
## 207  Sanger_isolates/48F.F.ab1 Sanger_isolates  48F.F.ab1       1157
## 208  Sanger_isolates/48R.R.ab1 Sanger_isolates  48R.R.ab1        200
## 209  Sanger_isolates/76F.F.ab1 Sanger_isolates  76F.F.ab1       1138
## 210  Sanger_isolates/76R.R.ab1 Sanger_isolates  76R.R.ab1       1168
## 211  Sanger_isolates/77F.F.ab1 Sanger_isolates  77F.F.ab1       1095
## 212  Sanger_isolates/77R.R.ab1 Sanger_isolates  77R.R.ab1       1111
## 213  Sanger_isolates/78F.F.ab1 Sanger_isolates  78F.F.ab1        944
## 214  Sanger_isolates/78R.R.ab1 Sanger_isolates  78R.R.ab1       1180
## 215  Sanger_isolates/79F.F.ab1 Sanger_isolates  79F.F.ab1        528
## 216  Sanger_isolates/79R.R.ab1 Sanger_isolates  79R.R.ab1        966
## 217  Sanger_isolates/80F.F.ab1 Sanger_isolates  80F.F.ab1        638
## 218  Sanger_isolates/80R.R.ab1 Sanger_isolates  80R.R.ab1        999
## 219  Sanger_isolates/81F.F.ab1 Sanger_isolates  81F.F.ab1        801
## 220  Sanger_isolates/81R.R.ab1 Sanger_isolates  81R.R.ab1       1031
## 221  Sanger_isolates/82F.F.ab1 Sanger_isolates  82F.F.ab1        891
## 222  Sanger_isolates/82R.R.ab1 Sanger_isolates  82R.R.ab1       1129
## 223  Sanger_isolates/83F.F.ab1 Sanger_isolates  83F.F.ab1        637
## 224  Sanger_isolates/83R.R.ab1 Sanger_isolates  83R.R.ab1        915
## 225  Sanger_isolates/84F.F.ab1 Sanger_isolates  84F.F.ab1        515
## 226  Sanger_isolates/84R.R.ab1 Sanger_isolates  84R.R.ab1       1088
## 227  Sanger_isolates/85F.F.ab1 Sanger_isolates  85F.F.ab1        525
## 228  Sanger_isolates/85R.R.ab1 Sanger_isolates  85R.R.ab1       1072
## 229  Sanger_isolates/86F.F.ab1 Sanger_isolates  86F.F.ab1        513
## 230  Sanger_isolates/86R.R.ab1 Sanger_isolates  86R.R.ab1       1104
## 231  Sanger_isolates/87F.F.ab1 Sanger_isolates  87F.F.ab1        699
## 232  Sanger_isolates/87R.R.ab1 Sanger_isolates  87R.R.ab1       1022
## 233  Sanger_isolates/88F.F.ab1 Sanger_isolates  88F.F.ab1        990
```

```
## 234  Sanger_isolates/88R.R.ab1 Sanger_isolates  88R.R.ab1        820
## 235  Sanger_isolates/89F.F.ab1 Sanger_isolates  89F.F.ab1        628
## 236  Sanger_isolates/89R.R.ab1 Sanger_isolates  89R.R.ab1        916
## 237  Sanger_isolates/90F.F.ab1 Sanger_isolates  90F.F.ab1        950
## 238  Sanger_isolates/90R.R.ab1 Sanger_isolates  90R.R.ab1       1182
## 239  Sanger_isolates/91F.F.ab1 Sanger_isolates  91F.F.ab1        973
## 240  Sanger_isolates/91R.R.ab1 Sanger_isolates  91R.R.ab1       1112
## 241  Sanger_isolates/92F.F.ab1 Sanger_isolates  92F.F.ab1        988
## 242  Sanger_isolates/92R.R.ab1 Sanger_isolates  92R.R.ab1       1127
## 243  Sanger_isolates/93F.F.ab1 Sanger_isolates  93F.F.ab1        933
## 244  Sanger_isolates/93R.R.ab1 Sanger_isolates  93R.R.ab1       1049
## 245  Sanger_isolates/94F.F.ab1 Sanger_isolates  94F.F.ab1        986
## 246  Sanger_isolates/94R.R.ab1 Sanger_isolates  94R.R.ab1        802
## 247  Sanger_isolates/95F.F.ab1 Sanger_isolates  95F.F.ab1       1050
## 248  Sanger_isolates/95R.R.ab1 Sanger_isolates  95R.R.ab1        911
## 249  Sanger_isolates/96F.F.ab1 Sanger_isolates  96F.F.ab1       1167
## 250  Sanger_isolates/96R.R.ab1 Sanger_isolates  96R.R.ab1       1125
## 251  Sanger_isolates/97F.F.ab1 Sanger_isolates  97F.F.ab1        915
## 252  Sanger_isolates/97R.R.ab1 Sanger_isolates  97R.R.ab1        993
## 253  Sanger_isolates/98F.F.ab1 Sanger_isolates  98F.F.ab1        713
## 254  Sanger_isolates/98R.R.ab1 Sanger_isolates  98R.R.ab1        716
## 255  Sanger_isolates/99F.F.ab1 Sanger_isolates  99F.F.ab1       1178
## 256  Sanger_isolates/99R.R.ab1 Sanger_isolates  99R.R.ab1       1116
##     trimmed.length trim.start trim.finish raw.secondary.peaks
## 1              314         24         337                 264
## 2                2        484         485                 237
## 3              610         20         629                  27
## 4              750         21         770                   8
## 5              573         20         592                  38
## 6              474         73         546                  20
## 7              625         14         638                  25
## 8              492         14         505                  12
## 9              557         17         573                  32
## 10             569         26         594                  29
## 11             624         14         637                  39
## 12             327          5         331                  74
## 13             589         14         602                  16
## 14             499          6         504                   7
## 15             624         11         634                  33
## 16             498          5         502                  32
## 17             609         12         620                  23
## 18             493         13         505                  26
## 19             604         10         613                  20
## 20             490         15         504                  42
## 21             610         11         620                  55
## 22             752         16         767                  24
## 23             621         11         631                   8
## 24             491         15         505                  19
## 25             319        143         461                 209
## 26             546         39         584                 238
```

```
## 27          363     11    373           58
## 28          730     17    746           38
## 29          356     17    372           55
## 30          730     19    748           29
## 31          344     23    366           26
## 32          310     14    323           16
## 33          408     85    492          265
## 34          825     19    843           23
## 35            0      0      0          145
## 36            0      0      0          136
## 37            0      0      0          147
## 38          754     16    769           25
## 39            0      0      0          223
## 40            1    341    341          243
## 41          168     22    189          177
## 42            0      0      0           99
## 43          214     16    229          201
## 44          832     21    852           14
## 45          566     17    582           19
## 46          594     15    608           24
## 47          237    192    428          204
## 48          463     18    480           35
## 49          231     34    264           56
## 50          122     17    138           23
## 51          355    120    474           81
## 52          462     40    501           86
## 53          343     37    379           31
## 54          457     54    510          150
## 55          365     11    375           55
## 56          204     17    220           38
## 57          498     25    522           40
## 58          307     14    320           42
## 59          560     20    579          116
## 60          829     18    846           39
## 61          594     23    616           47
## 62          729     19    747           28
## 63          354     22    375           57
## 64          204     18    221           43
## 65           72     83    154          174
## 66          797     19    815           16
## 67          353     22    374           54
## 68          730     15    744           22
## 69          225    277    501          264
## 70           38     10     47          232
## 71          559     23    581           17
## 72          707     18    724           26
## 73          423     17    439            6
## 74          753     17    769           23
## 75          256     13    268          271
## 76          770     19    788           14
```

```
## 77                    294          85          378                    104
## 78                    606          18          623                     16
## 79                    407          78          484                    121
## 80                    719          14          732                     22
## 81                    408          85          492                    156
## 82                    718          19          736                     29
## 83                    526          12          537                     38
## 84                    753          16          768                     22
## 85                    295          85          379                     88
## 86                    737          20          756                      8
## 87                    295          85          379                    154
## 88                    606          19          624                      7
## 89                     68          87          154                    161
## 90                    730          18          747                     40
## 91                    295          81          375                    124
## 92                    715          19          733                     26
## 93                    404          86          489                    131
## 94                    482          30          511                     36
## 95                    351          26          376                    149
## 96                    732          18          749                     23
## 97                     68          86          153                    160
## 98                    617          18          634                      5
## 99                    352          27          378                    112
## 100                   299          12          310                    135
## 101                   111          20          130                    223
## 102                   612          68          679                     32
## 103                   759           7          765                     17
## 104                   727          18          744                     33
## 105                   706          14          719                     28
## 106                   536          11          546                     25
## 107                   287         240          526                    242
## 108                   847          20          866                    111
## 109                   403          21          423                    286
## 110                   589          17          605                     21
## 111                   110          17          126                    313
## 112                   588          17          604                     19
## 113                   100          31          130                    105
## 114                   589          15          603                     11
## 115                   713          18          730                     11
## 116                   830          16          845                     10
## 117                   695          17          711                     18
## 118                   839          14          852                      9
## 119                   679          16          694                     32
## 120                   818          22          839                     20
## 121                    48          74          121                    115
## 122                   506           6          511                     24
## 123                   712          15          726                     34
## 124                   539          15          553                     33
## 125                   782          17          798                     22
## 126                   840          10          849                     14
```

```
## 127          712     18      729         41
## 128          890     11      900         15
## 129          678     21      698         16
## 130          788      8      795         43
## 131          623     12      634         49
## 132          499      8      506          9
## 133          534     18      551         12
## 134          693     14      706         13
## 135          708     18      725         34
## 136          689     18      706          7
## 137          345    325      669        461
## 138          830     15      844         22
## 139          703      6      708         47
## 140          319     10      328        267
## 141            4    388      391        438
## 142          690     17      706         28
## 143          629     11      639         77
## 144          516     15      530         25
## 145          592     18      609         31
## 146            1    368      368        243
## 147          713      8      720          9
## 148          687     20      706         32
## 149          705     15      719         22
## 150          922     18      939         13
## 151            0      0        0        331
## 152          440     13      452        118
## 153            1    603      603        431
## 154           28     14       41        361
## 155            0      0        0        380
## 156            0      0        0        416
## 157          718     15      732         13
## 158          963     17      979         21
## 159           75     20       94        411
## 160          465     11      475         48
## 161          561     74      634        107
## 162          748     19      766         30
## 163          697     14      710         24
## 164          610     13      622          5
## 165          539     39      577         48
## 166          349     15      363         31
## 167          245     34      278        262
## 168          619     17      635         14
## 169          588      7      594         44
## 170          738      1      738         24
## 171          615     14      628         83
## 172          716     11      726         14
## 173          621     19      639         25
## 174          582     21      602         30
## 175          642     25      666         24
## 176          831      9      839         33
```

```
## 177         586      11         596             24
## 178         616       9         624              7
## 179         360      99         458            126
## 180         761      23         783             35
## 181         560      21         580             29
## 182         763      10         772             19
## 183         512      16         527             33
## 184         643      19         661             24
## 185         633      21         653             25
## 186         727      16         742              4
## 187         519      10         528             63
## 188         411      17         427             20
## 189         342      15         356             31
## 190          72      15          86            207
## 191         645      10         654             29
## 192         783      18         800             11
## 193         713      14         726             71
## 194         747      17         763             12
## 195         629      11         639             44
## 196         586      14         599             54
## 197         641      15         655             17
## 198         828      14         841             22
## 199         499      23         521             56
## 200         740      15         754             14
## 201         585      16         600             39
## 202         317      34         350            199
## 203         550      17         566             31
## 204         700      30         729             29
## 205         547      20         566             28
## 206         314      13         326             33
## 207         524      14         537             57
## 208           0       0           0            129
## 209         706       9         714             11
## 210         839      14         852             14
## 211         215      46         260            367
## 212         636      33         668            268
## 213         633      14         646             30
## 214         716      28         743             12
## 215         225      15         239            107
## 216         751      23         773             12
## 217         441      76         516            114
## 218         275      26         300             47
## 219         567       7         573             64
## 220         796      21         816             17
## 221         556      10         565             28
## 222         710      17         726             15
## 223         594      21         614             12
## 224         709      33         741             25
## 225         501      14         514              0
## 226         769      24         792             30
```

```
## 227                291          10          300                   27
## 228                729          17          745                   12
## 229                277          22          298                    4
## 230                819          21          839                   26
## 231                474          25          498                   41
## 232                713          17          729                    9
## 233                617           8          624                   67
## 234                655          19          673                   42
## 235                 40         139          178                  220
## 236                 30          30           59                  340
## 237                283          58          340                  105
## 238                901          19          919                   13
## 239                709           9          717                   24
## 240                654          18          671                   16
## 241                614          16          629                   13
## 242                911          10          920                   10
## 243                705          17          721                   12
## 244                706          15          720                   10
## 245                199          76          274                  280
## 246                581          23          603                   33
## 247                 81          70          150                  205
## 248                198          29          226                   33
## 249                610          14          623                   46
## 250                723          20          742                   13
## 251                476         217          692                  126
## 252                 85          55          139                  204
## 253                534          15          548                   38
## 254                595          19          613                    3
## 255                680          17          696                   28
## 256                909          17          925                   14
##     trimmed.secondary.peaks raw.mean.quality trimmed.mean.quality
## 1                        68         23.52626             28.95860
## 2                         1         17.29835             41.00000
## 3                         2         45.56604             51.27049
## 4                         0         47.80229             52.50400
## 5                         5         38.44748             45.30890
## 6                         1         41.82790             50.24684
## 7                         0         44.18926             52.12320
## 8                         1         46.71632             52.29472
## 9                         6         42.88615             50.83124
## 10                        1         42.21843             48.74868
## 11                        0         43.04356             51.55609
## 12                        8         44.62283             53.20489
## 13                        0         45.51059             51.64686
## 14                        3         46.27200             51.16836
## 15                        0         42.58244             51.13141
## 16                        4         43.72024             51.44309
## 17                        0         44.96687             51.40558
## 18                        1         45.29670             52.06288
## 19                        0         43.70712             51.82616
```

```
## 20                      3     44.36121            51.89796
## 21                      1     41.73789            51.63607
## 22                      0     46.10872            52.54122
## 23                      1     46.69818            51.25282
## 24                      4     44.35714            49.69654
## 25                    122     19.52190            20.86834
## 26                    202     21.88141            22.33516
## 27                      2     41.69990            50.81267
## 28                      4     45.30661            51.90548
## 29                      2     44.29032            50.97472
## 30                      5     44.81320            51.05342
## 31                      1     42.53370            48.79651
## 32                      0     47.19084            57.95161
## 33                     65     25.31256            33.45588
## 34                      0     46.69182            52.10182
## 35                      0     11.50000                  NA
## 36                      0      9.68500                  NA
## 37                      0      9.54000                  NA
## 38                      0     46.45817            51.85942
## 39                      0     13.90941                  NA
## 40                      0     16.65982            51.00000
## 41                      0     33.66131            48.80952
## 42                      0     13.94574                  NA
## 43                     29     26.37595            35.11682
## 44                      0     47.07706            52.07332
## 45                      1     50.60893            56.80212
## 46                      0     47.13833            50.88047
## 47                     41     26.01257            33.25738
## 48                     11     44.81257            49.79482
## 49                     20     40.38725            44.09957
## 50                      5     40.43455            39.86885
## 51                     21     36.43493            43.17465
## 52                     12     39.42961            47.54762
## 53                      6     39.91544            44.66181
## 54                     69     35.06421            39.51204
## 55                      0     40.40994            51.49041
## 56                      1     43.90269            50.26961
## 57                      2     42.61054            48.40161
## 58                      0     41.78200            50.64169
## 59                      0     37.81471            51.78393
## 60                      9     45.50294            50.45838
## 61                      1     40.44714            48.74242
## 62                      3     44.63526            51.66255
## 63                      1     42.36053            50.97740
## 64                      0     42.45929            50.63725
## 65                     11     28.17019            35.50000
## 66                      0     46.61232            51.95107
## 67                      3     42.39801            50.27479
## 68                      5     45.96314            51.26849
## 69                     63     19.39213            23.85778
```

```
## 70                       9          14.86667              23.94737
## 71                       1          45.83409              49.19857
## 72                       3          43.60435              51.69731
## 73                       1          45.42436              48.10875
## 74                       1          47.12354              51.25365
## 75                      84          26.38418              27.64062
## 76                       1          47.26456              52.04545
## 77                      47          30.14821              32.12585
## 78                       0          47.72393              51.65842
## 79                      64          30.43121              33.80344
## 80                       0          46.96162              51.80807
## 81                      66          29.17797              33.41176
## 82                       0          44.49638              53.04735
## 83                       1          43.10359              48.40684
## 84                       3          44.52515              51.00398
## 85                      41          31.71031              34.21695
## 86                       0          47.69884              52.05020
## 87                      49          29.23843              33.68136
## 88                       0          46.78559              52.96205
## 89                      11          29.88686              34.75000
## 90                       0          45.77387              52.00548
## 91                      44          30.51278              33.48136
## 92                       0          45.26036              52.20839
## 93                      10          39.76145              53.92079
## 94                       7          45.59307              50.04357
## 95                      61          30.09487              32.08547
## 96                       0          44.83675              52.36885
## 97                      14          29.22585              34.32353
## 98                       0          47.66243              52.77958
## 99                      54          31.28186              31.79830
## 100                      3          38.86575              52.85284
## 101                      3          26.53713              47.02703
## 102                      9          43.79380              48.55229
## 103                      0          45.57021              51.87352
## 104                      1          45.90696              52.61073
## 105                      2          46.25922              51.52691
## 106                      2          49.07506              57.05784
## 107                     64          26.86437              30.41812
## 108                     22          39.32750              48.58796
## 109                     93          23.98364              30.43921
## 110                      1          46.42845              52.27844
## 111                     31          26.95376              32.40909
## 112                      1          45.30882              51.76531
## 113                     19          31.10756              33.68000
## 114                      3          48.83965              52.37012
## 115                      0          46.89791              52.07293
## 116                      2          46.71604              51.49157
## 117                      7          45.79581              51.01007
## 118                      5          49.07415              50.99046
## 119                      4          44.54502              50.10457
```

```
## 120                       2           46.13967             50.09169
## 121                       3           37.86280             50.04167
## 122                      12           44.37313             48.00988
## 123                       3           44.11610             50.15730
## 124                       3           49.96474             56.99629
## 125                       3           47.09756             51.26854
## 126                       1           46.81638             51.57976
## 127                       0           44.17473             52.06882
## 128                       2           46.29256             51.57191
## 129                       0           45.55676             51.15782
## 130                       1           43.72099             50.80584
## 131                       0           42.37877             51.09952
## 132                       0           45.78534             51.83768
## 133                       0           45.72338             51.79213
## 134                       0           47.28150             52.48052
## 135                       0           44.07912             51.60311
## 136                       0           48.79946             53.03048
## 137                     243           13.87130             13.66087
## 138                       1           45.91261             52.04699
## 139                       0           43.39892             50.79232
## 140                     100           27.14393             29.44201
## 141                       4           13.86864             32.25000
## 142                       1           46.50494             51.06957
## 143                      53           34.43218             37.30366
## 144                       4           42.84799             51.32558
## 145                       2           44.51629             52.33277
## 146                       1           14.18699             45.00000
## 147                       2           46.45987             51.45302
## 148                       3           46.46591             51.60408
## 149                       1           45.02753             51.66099
## 150                       3           47.18482             51.46095
## 151                       0           13.25934                   NA
## 152                      23           38.89889             48.61364
## 153                       0           14.32338             42.00000
## 154                       0           15.65925             46.64286
## 155                       0           12.86242                   NA
## 156                       0           12.37633                   NA
## 157                       1           45.42989             52.26741
## 158                       2           46.64190             52.01454
## 159                       2           17.59757             39.37333
## 160                      20           43.77935             46.73763
## 161                      28           33.30795             39.86631
## 162                       6           45.12286             49.24465
## 163                       0           43.64877             52.24821
## 164                       0           51.26779             57.74098
## 165                       5           41.28306             48.91651
## 166                       5           47.21540             51.14900
## 167                      77           26.96061             30.80408
## 168                       3           45.81703             50.77544
## 169                       0           42.57407             52.03741
```

```
## 170                 0     44.61325     52.06967
## 171                 3     40.12092     50.23902
## 172                 3     47.72076     51.59916
## 173                 1     44.13673     52.07729
## 174                 2     45.81655     51.86770
## 175                 1     45.08815     52.33645
## 176                 1     43.88818     52.10469
## 177                 0     44.15400     51.71843
## 178                 0     47.25962     50.52922
## 179                51     32.44124     35.23889
## 180                 6     44.07605     50.28252
## 181                 1     45.26030     51.95179
## 182                 0     45.71769     52.24771
## 183                 0     41.94208     48.58203
## 184                 1     43.80748     49.44946
## 185                 0     45.64942     52.08057
## 186                 0     47.88824     52.20083
## 187                 3     45.16516     52.90366
## 188                 4     45.52700     50.73966
## 189                 0     42.61411     48.48538
## 190                 0     23.30952     45.31944
## 191                 0     46.01811     52.24031
## 192                 0     47.42912     52.64112
## 193                 1     40.15228     50.86816
## 194                 0     47.17663     52.30254
## 195                 2     42.96177     49.65978
## 196                 2     45.09729     55.10922
## 197                 1     45.53771     52.27769
## 198                 2     45.97950     52.40580
## 199                 2     43.78778     54.23246
## 200                 0     46.84308     51.78784
## 201                 0     43.03297     51.89744
## 202                30     25.50342     29.71609
## 203                15     41.75907     44.21818
## 204                 9     41.05775     46.70000
## 205                 1     43.88977     49.17733
## 206                 1     42.77949     51.51592
## 207                 0     39.53012     49.66985
## 208                 0      9.80500           NA
## 209                 0     44.99567     52.06516
## 210                 2     43.39237     50.66031
## 211                68     20.19143     23.00465
## 212               195     25.31847     25.66195
## 213                 4     48.50936     55.63507
## 214                 1     46.05228     51.41480
## 215                 2     40.14205     47.48889
## 216                 2     48.06082     51.01731
## 217                72     32.29844     35.15873
## 218                 4     41.25222     47.86909
## 219                 1     48.68897     58.84656
```

```
## 220                    2         48.70281             52.08794
## 221                    3         49.28101             57.92086
## 222                    0         48.26678             53.06479
## 223                    5         45.04702             46.95286
## 224                   11         45.16576             47.93794
## 225                    0         51.21470             52.21557
## 226                    4         44.86447             49.32900
## 227                    2         45.89184             51.19931
## 228                    2         48.61860             52.56927
## 229                    1         48.41942             51.59567
## 230                    1         47.16937             52.70574
## 231                    0         42.68714             51.18987
## 232                    0         47.34732             49.84853
## 233                   13         42.83585             50.72123
## 234                    0         45.38929             51.47939
## 235                    4         23.82194             38.97500
## 236                    0         20.64130             50.10000
## 237                   26         34.88655             41.56184
## 238                    1         46.73662             51.73030
## 239                    0         46.37755             51.01693
## 240                    0         47.77907             53.06269
## 241                    0         48.38153             52.37296
## 242                    0         48.85915             52.58836
## 243                    0         47.27079             50.78440
## 244                    3         48.34414             51.65864
## 245                   71         28.73834             34.01005
## 246                   13         42.83602             45.78657
## 247                    2         29.74430             45.71605
## 248                    3         41.85137             46.61111
## 249                    2         40.89957             49.95738
## 250                    4         46.03530             51.77593
## 251                   54         33.62568             37.63445
## 252                   14         28.39196             32.38824
## 253                    1         43.49930             51.18539
## 254                    0         50.22981             52.82689
## 255                    4         44.20185             51.02059
## 256                    2         46.10993             51.00880
##     raw.min.quality trimmed.min.quality
## 1                 2                   8
## 2                 2                  41
## 3                 2                  15
## 4                 2                  10
## 5                 2                   2
## 6                 2                  23
## 7                 2                   7
## 8                 2                   7
## 9                 2                   7
## 10                2                   2
## 11                2                   7
## 12                2                   7
```

```
## 13                  2                    7
## 14                  2                    9
## 15                  2                    7
## 16                  2                   10
## 17                  2                    7
## 18                  2                   15
## 19                  2                    7
## 20                  2                   10
## 21                  2                   10
## 22                  2                    2
## 23                  7                    7
## 24                  2                    6
## 25                  2                    7
## 26                  2                    2
## 27                  4                   12
## 28                  3                    3
## 29                  2                    9
## 30                  4                    8
## 31                  4                   16
## 32                  5                    9
## 33                  2                   10
## 34                  3                    9
## 35                  4                   NA
## 36                  3                   NA
## 37                  3                   NA
## 38                  5                   14
## 39                  5                   NA
## 40                  6                   51
## 41                  5                   20
## 42                  5                   NA
## 43                  2                    7
## 44                  2                   22
## 45                  5                    5
## 46                  7                   22
## 47                  2                    8
## 48                  5                    7
## 49                  5                    5
## 50                  5                    8
## 51                  2                    7
## 52                  2                    6
## 53                  4                   12
## 54                  4                    4
## 55                  2                   12
## 56                  3                    6
## 57                  5                   15
## 58                  5                    7
## 59                  5                   20
## 60                  3                   10
## 61                  4                   15
## 62                  5                   14
```

```
## 63                    5                    20
## 64                    5                     6
## 65                    2                    12
## 66                    5                     9
## 67                    4                     9
## 68                    5                     8
## 69                    2                     5
## 70                    5                     9
## 71                    4                    14
## 72                    5                    14
## 73                    5                     8
## 74                    5                    14
## 75                    4                     8
## 76                    5                     9
## 77                    2                     8
## 78                    5                     9
## 79                    5                    10
## 80                    3                     9
## 81                    2                    10
## 82                    2                     9
## 83                    3                    20
## 84                    2                    14
## 85                    2                    10
## 86                    4                     8
## 87                    5                    12
## 88                    4                     8
## 89                    6                    12
## 90                    4                     8
## 91                    2                    10
## 92                    4                     7
## 93                    2                    31
## 94                    2                     7
## 95                    2                     7
## 96                    2                     8
## 97                    6                    12
## 98                    2                    14
## 99                    3                     7
## 100                   5                     9
## 101                   6                    14
## 102                   5                    12
## 103                   5                     8
## 104                   5                    12
## 105                   5                     5
## 106                   5                     5
## 107                   2                     8
## 108                   5                     8
## 109                   2                     7
## 110                   5                    10
## 111                   5                    10
## 112                   2                    14
```

```
## 113                5                   10
## 114                5                    8
## 115                4                   11
## 116                2                    2
## 117                5                    5
## 118                4                    4
## 119                3                    5
## 120                5                    5
## 121                2                   24
## 122                2                    8
## 123                2                    4
## 124                5                    8
## 125                5                    6
## 126                5                    9
## 127                5                   20
## 128                2                   18
## 129                2                    2
## 130                5                    5
## 131                5                   20
## 132                2                    5
## 133                6                   11
## 134                4                    9
## 135                5                   14
## 136                5                    9
## 137                5                    5
## 138                3                   10
## 139                5                    5
## 140                4                   10
## 141                5                   19
## 142                2                   12
## 143                2                    7
## 144                2                    7
## 145                6                   20
## 146                5                   45
## 147                6                    8
## 148                5                    5
## 149                4                    9
## 150                4                    4
## 151                5                   NA
## 152                5                    8
## 153                5                   42
## 154                5                   15
## 155                5                   NA
## 156                5                   NA
## 157                5                    8
## 158                5                    8
## 159                2                    8
## 160                2                    6
## 161                2                   10
## 162                2                    2
```

```
## 163                    2                    20
## 164                    2                    12
## 165                    2                     7
## 166                    2                     2
## 167                    2                     8
## 168                    2                     6
## 169                    2                     7
## 170                    2                    10
## 171                    2                    12
## 172                    2                     8
## 173                    2                    20
## 174                    2                     8
## 175                    2                    10
## 176                    2                     2
## 177                    2                    15
## 178                    2                     2
## 179                    2                    10
## 180                    2                     7
## 181                    2                    20
## 182                    2                     2
## 183                    2                    14
## 184                    2                     6
## 185                    2                    20
## 186                    2                     2
## 187                    2                     9
## 188                    2                     2
## 189                    2                     7
## 190                    5                    10
## 191                    2                     7
## 192                    2                    14
## 193                    2                    20
## 194                    2                    20
## 195                    2                     6
## 196                    2                     2
## 197                    2                    14
## 198                    2                     8
## 199                    2                     6
## 200                    2                     6
## 201                    2                    14
## 202                    2                     2
## 203                    2                     8
## 204                    2                     9
## 205                    6                    15
## 206                    6                     6
## 207                    2                    20
## 208                    3                    NA
## 209                    2                     9
## 210                    2                     2
## 211                    2                     7
## 212                    2                     8
```

```
## 213                  2                    2
## 214                  2                    7
## 215                  2                    2
## 216                  2                    2
## 217                  6                    8
## 218                  2                    7
## 219                  2                   13
## 220                  2                   10
## 221                  2                   14
## 222                  2                   12
## 223                  6                    7
## 224                  2                   14
## 225                  2                   15
## 226                  2                    8
## 227                  2                   14
## 228                  2                   12
## 229                  7                   12
## 230                  2                    9
## 231                  2                   20
## 232                  2                   15
## 233                  2                    7
## 234                  2                   22
## 235                  2                   14
## 236                  2                   40
## 237                  2                   10
## 238                  2                    2
## 239                  2                   14
## 240                  2                    7
## 241                  2                   15
## 242                  2                   13
## 243                  2                   14
## 244                  2                    7
## 245                  2                   13
## 246                  6                   10
## 247                  2                   21
## 248                  2                    2
## 249                  2                    2
## 250                  2                    6
## 251                  2                   10
## 252                  2                    2
## 253                  2                   15
## 254                  2                    8
## 255                  2                    7
## 256                  2                    2
```

```
p<- ggplot(sf$summaries, aes(x = folder.name, y = raw.mean.quality)) + geom_b
oxplot()
p <- ggplot(sf$summaries, aes(x = folder.name, y = trimmed.mean.quality)) + g
eom_boxplot()
p <- ggplot(sf$summaries, aes(x = folder.name, y = trimmed.secondary.peaks))
```

```r
+ geom_boxplot() + geom_hline(yintercept = 2, linetype = 3, colour = 'red')
p <- ggplot(sf$summaries, aes(x = trimmed.mean.quality, y = trimmed.secondary
.peaks)) + geom_point()

# Make consensus by merging reads
fwd = readsangerseq("Sanger_isolates/13F.F.ab1")
rev = readsangerseq("Sanger_isolates/13R.R.ab1")
fwd = primarySeq(fwd)
rev = primarySeq(rev)
rev = reverseComplement(rev)
reads = DNAStringSet(c(as.character(fwd), as.character(rev)))
names(reads) = c('fwd', 'rev')
merged.reads = merge.reads(reads)
```

```
## [1] "Aligning reads"
## [1] "Calling consensus sequence"
## [1] "Calculating differences between reads and consensus"
```

```r
names(merged.reads)
```

```
## [1] "consensus"              "alignment"
## [3] "differences"            "distance.matrix"
## [5] "dendrogram"             "indels"
## [7] "stop.codons"            "secondary.peak.columns"
```

```r
merged.reads
```

```
## $consensus
##   1283-letter "DNAString" instance
## seq: GATGCAATCTACAGATTCTGCATACAGGCTACTA...TCGCCTACATGAAGTNCGGAATCGCTAGTAAT
C
##
## $alignment
##   A DNAStringSet instance of length 3
##     width seq                                          names
## [1]  1283 GATGCAATCTACAGATTCTGC...--------------------- fwd
## [2]  1283 ---------------------...GTNCGGAATCGCTAGTAATC rev
## [3]  1283 GATGCAATCTACAGATTCTGC...GTNCGGAATCGCTAGTAATC consensus
##
## $differences
##   name pairwise.diffs.to.consensus unused.chars
## 1  fwd                          60            0
## 2  rev                          67            0
##
## $distance.matrix
##           fwd       rev
## fwd 0.0000000 0.2491807
## rev 0.2491807 0.0000000
## attr(,"correction")
## [1] "Jukes-Cantor"
##
```

```
## $dendrogram
## 'dendrogram' with 2 branches and 2 members total, at height 0.1245903
##
## $indels
## NULL
##
## $stop.codons
## NULL
##
## $secondary.peak.columns
## NULL
##
## attr(,"class")
## [1] "merged.read"
```

```
merged.reads$consensus
```

```
##   1283-letter "DNAString" instance
## seq: GATGCAATCTACAGATTCTGCATACAGGCTACTA...TCGCCTACATGAAGTNCGGAATCGCTAGTAAT
C
```

```
BrowseSeqs(merged.reads$alignment)
merged.reads$secondary.peak.columns
```

```
## NULL
```

```
merged.reads$differences
```

```
##   name pairwise.diffs.to.consensus unused.chars
## 1  fwd                          60            0
## 2  rev                          67            0
```

```
write.dna(merged.reads,file="Sanger_isolates/13.fasta",format='fasta',nbcol =
-1, colsep = "", colw = 10000000)

fwd = readsangerseq("Sanger_isolates/30F.F.ab1")
rev = readsangerseq("Sanger_isolates/30R.R.ab1")
fwd = primarySeq(fwd)
rev = primarySeq(rev)
rev = reverseComplement(rev)
reads = DNAStringSet(c(as.character(fwd), as.character(rev)))
names(reads) = c('fwd', 'rev')
merged.reads = merge.reads(reads)
```

```
## [1] "Aligning reads"
## [1] "Calling consensus sequence"
## [1] "Calculating differences between reads and consensus"
```

```
names(merged.reads)
```

```
## [1] "consensus"              "alignment"
## [3] "differences"            "distance.matrix"
```

```
## [5] "dendrogram"              "indels"
## [7] "stop.codons"             "secondary.peak.columns"
```

```
merged.reads
```

```
## $consensus
##   1285-letter "DNAString" instance
## seq: GGCAGCTTGCTGCTTTGCTGACGAGTGGCGGACG...CGACTCCATGAAGTCGGAATCGCTGTAATCGT
N
##
## $alignment
##   A DNAStringSet instance of length 3
##     width seq                                               names
## [1]  1285 GGCAGCTTGCTGCTTTGCTGA...--------------------- fwd
## [2]  1285 ---------------------...TCGGAATCGCTGTAATCGTN rev
## [3]  1285 GGCAGCTTGCTGCTTTGCTGA...TCGGAATCGCTGTAATCGTN consensus
##
## $differences
##   name pairwise.diffs.to.consensus unused.chars
## 1  fwd                          40            0
## 2  rev                          35            0
##
## $distance.matrix
##             fwd        rev
## fwd 0.00000000 0.06081996
## rev 0.06081996 0.00000000
## attr(,"correction")
## [1] "Jukes-Cantor"
##
## $dendrogram
## 'dendrogram' with 2 branches and 2 members total, at height 0.03040998
##
## $indels
## NULL
##
## $stop.codons
## NULL
##
## $secondary.peak.columns
## NULL
##
## attr(,"class")
## [1] "merged.read"
```

```
merged.reads$consensus
```

```
##   1285-letter "DNAString" instance
## seq: GGCAGCTTGCTGCTTTGCTGACGAGTGGCGGACG...CGACTCCATGAAGTCGGAATCGCTGTAATCGT
N
```

```r
BrowseSeqs(merged.reads$alignment)
merged.reads$secondary.peak.columns

## NULL

merged.reads$differences

##   name pairwise.diffs.to.consensus unused.chars
## 1  fwd                          40            0
## 2  rev                          35            0

write.dna(merged.reads,file="Sanger_isolates/30.fasta",format='fasta',nbcol =
-1, colsep = "", colw = 10000000)

fwd = readsangerseq("Sanger_isolates/95F.F.ab1")
rev = readsangerseq("Sanger_isolates/95R.R.ab1")
fwd = primarySeq(fwd)
rev = primarySeq(rev)
rev = reverseComplement(rev)
reads = DNAStringSet(c(as.character(fwd), as.character(rev)))
names(reads) = c('fwd', 'rev')
merged.reads = merge.reads(reads)

## [1] "Aligning reads"
## [1] "Calling consensus sequence"
## [1] "Calculating differences between reads and consensus"

names(merged.reads)

## [1] "consensus"                "alignment"
## [3] "differences"              "distance.matrix"
## [5] "dendrogram"               "indels"
## [7] "stop.codons"              "secondary.peak.columns"

merged.reads

## $consensus
##   1289-letter "DNAString" instance
## seq: NNGCTCTTATGAGGTTAGCGGCGGACGGGTGAGT...TACATGAAGCTGGAATCGCTAGTAATCGCGAT
C
##
## $alignment
##   A DNAStringSet instance of length 3
##     width seq                                               names
## [1]  1289 NNGCTCTTATGAGGTTAGCGG...--------------------- fwd
## [2]  1289 ---------------------...AATCGCTAGTAATCGCGATC rev
## [3]  1289 NNGCTCTTATGAGGTTAGCGG...AATCGCTAGTAATCGCGATC consensus
##
## $differences
##   name pairwise.diffs.to.consensus unused.chars
## 1  fwd                          66            0
## 2  rev                          62            0
```

```
##
## $distance.matrix
##              fwd        rev
## fwd 0.00000000 0.09751936
## rev 0.09751936 0.00000000
## attr(,"correction")
## [1] "Jukes-Cantor"
##
## $dendrogram
## 'dendrogram' with 2 branches and 2 members total, at height 0.04875968
##
## $indels
## NULL
##
## $stop.codons
## NULL
##
## $secondary.peak.columns
## NULL
##
## attr(,"class")
## [1] "merged.read"
```

```
merged.reads$consensus
```

```
##   1289-letter "DNAString" instance
## seq: NNGCTCTTATGAGGTTAGCGGCGGACGGGTGAGT...TACATGAAGCTGGAATCGCTAGTAATCGCGAT
C
```

```
BrowseSeqs(merged.reads$alignment)
merged.reads$secondary.peak.columns
```

```
## NULL
```

```
merged.reads$differences
```

```
##   name pairwise.diffs.to.consensus unused.chars
## 1  fwd                          66            0
## 2  rev                          62            0
```

```
write.dna(merged.reads,file="Sanger_isolates/95.fasta",format='fasta',nbcol =
-1, colsep = "", colw = 10000000)

fwd = readsangerseq("Sanger_isolates/98F.F.ab1")
rev = readsangerseq("Sanger_isolates/98R.R.ab1")
fwd = primarySeq(fwd)
rev = primarySeq(rev)
rev = reverseComplement(rev)
reads = DNAStringSet(c(as.character(fwd), as.character(rev)))
names(reads) = c('fwd', 'rev')
merged.reads = merge.reads(reads)
```

```
## [1] "Aligning reads"
## [1] "Calling consensus sequence"
## [1] "Calculating differences between reads and consensus"
```

```
names(merged.reads)
```

```
## [1] "consensus"              "alignment"
## [3] "differences"            "distance.matrix"
## [5] "dendrogram"             "indels"
## [7] "stop.codons"            "secondary.peak.columns"
```

```
merged.reads
```

```
## $consensus
##   737-letter "DNAString" instance
## seq: NNAGCGAGKRRCSWCSGCKGMKTRRSWCSKGKAG...CWYSMMKWCSGARTYSCWTGYAMKCKMGRTYA
G
##
## $alignment
##   A DNAStringSet instance of length 3
##     width seq                                          names
## [1]   737 NNAGCGAGTGGCGACGGCTGA...NCCCATGCACGCTAGGTT-- fwd
## [2]   737 -------GGAACCTCCGCGGC...TTGCTNGTAATCGCGATCAG rev
## [3]   737 NNAGCGAGKRRCSWCSGCKGM...TYSCWTGYAMKCKMGRTYAG consensus
##
## $differences
##   name pairwise.diffs.to.consensus unused.chars
## 1  fwd                         399            0
## 2  rev                         394            0
##
## $distance.matrix
##          fwd      rev
## fwd 0.000000 1.029787
## rev 1.029787 0.000000
## attr(,"correction")
## [1] "Jukes-Cantor"
##
## $dendrogram
## 'dendrogram' with 2 branches and 2 members total, at height 0.5148934
##
## $indels
## NULL
##
## $stop.codons
## NULL
##
## $secondary.peak.columns
## NULL
##
## attr(,"class")
## [1] "merged.read"
```

```r
merged.reads$consensus

##   737-letter "DNAString" instance
## seq: NNAGCGAGKRRCSWCSGCKGMKTRRSWCSKGKAG...CWYSMMKWCSGARTYSCWTGYAMKCKMGRTYA
G

BrowseSeqs(merged.reads$alignment)
merged.reads$secondary.peak.columns

## NULL

merged.reads$differences

##   name pairwise.diffs.to.consensus unused.chars
## 1  fwd                         399            0
## 2  rev                         394            0

write.dna(merged.reads,file="Sanger_isolates/98.fasta",format='fasta',nbcol =
-1, colsep = "", colw = 10000000)

fwd = readsangerseq("Sanger_isolates/137F.F.ab1")
rev = readsangerseq("Sanger_isolates/137R.R.ab1")
fwd = primarySeq(fwd)
rev = primarySeq(rev)
rev = reverseComplement(rev)
reads = DNAStringSet(c(as.character(fwd), as.character(rev)))
names(reads) = c('fwd', 'rev')
merged.reads = merge.reads(reads)

## [1] "Aligning reads"
## [1] "Calling consensus sequence"
## [1] "Calculating differences between reads and consensus"

names(merged.reads)

## [1] "consensus"             "alignment"
## [3] "differences"           "distance.matrix"
## [5] "dendrogram"            "indels"
## [7] "stop.codons"           "secondary.peak.columns"

merged.reads

## $consensus
##   1276-letter "DNAString" instance
## seq: GTTGGAGAGTTGCGAACGGGTGAGTAACGCGTAG...ATGAAGTCGGAATCGCTAGTAATCGCGGATCA
G
##
## $alignment
##   A DNAStringSet instance of length 3
##     width seq                                          names
## [1]  1276 GTTGGAGAGTTGCGAACGGGT...--------------------- fwd
## [2]  1276 ---------------------...CGCTAGTAATCGCGGATCAG rev
```

```
## [3]  1276 GTTGGAGAGTTGCGAACGGGT...CGCTAGTAATCGCGGATCAG consensus
##
## $differences
##   name pairwise.diffs.to.consensus unused.chars
## 1  fwd                           6            0
## 2  rev                           6            0
##
## $distance.matrix
##            fwd        rev
## fwd 0.00000000 0.03922462
## rev 0.03922462 0.00000000
## attr(,"correction")
## [1] "Jukes-Cantor"
##
## $dendrogram
## 'dendrogram' with 2 branches and 2 members total, at height 0.01961231
##
## $indels
## NULL
##
## $stop.codons
## NULL
##
## $secondary.peak.columns
## NULL
##
## attr(,"class")
## [1] "merged.read"
```

```
merged.reads$consensus
```

```
##   1276-letter "DNAString" instance
## seq: GTTGGAGAGTTGCGAACGGGTGAGTAACGCGTAG...ATGAAGTCGGAATCGCTAGTAATCGCGGATCA
G
```

```
BrowseSeqs(merged.reads$alignment)
merged.reads$secondary.peak.columns
```

```
## NULL
```

```
merged.reads$differences
```

```
##   name pairwise.diffs.to.consensus unused.chars
## 1  fwd                           6            0
## 2  rev                           6            0
```

```
write.dna(merged.reads,file="Sanger_isolates/137.fasta",format='fasta',nbcol
= -1, colsep = "", colw = 10000000)

fwd = readsangerseq("Sanger_isolates/138F.F.ab1")
rev = readsangerseq("Sanger_isolates/138R.R.ab1")
```

```r
fwd = primarySeq(fwd)
rev = primarySeq(rev)
rev = reverseComplement(rev)
reads = DNAStringSet(c(as.character(fwd), as.character(rev)))
names(reads) = c('fwd', 'rev')
merged.reads = merge.reads(reads)

## [1] "Aligning reads"
## [1] "Calling consensus sequence"
## [1] "Calculating differences between reads and consensus"

names(merged.reads)

## [1] "consensus"               "alignment"
## [3] "differences"             "distance.matrix"
## [5] "dendrogram"              "indels"
## [7] "stop.codons"             "secondary.peak.columns"

merged.reads

## $consensus
##   1297-letter "DNAString" instance
## seq: TGACTGCTCTGCCTCCTGATTGAATAATCGTTTG...TGAAGCCGGAATCGCTAGTAATCGCGGATCAG
C
##
## $alignment
##   A DNAStringSet instance of length 3
##     width seq                                               names
## [1]  1297 TGACTGCTCTGCCTCCTGATT...--------------------- fwd
## [2]  1297 ---------------------...GCTAGTAATCGCGGATCAGC rev
## [3]  1297 TGACTGCTCTGCCTCCTGATT...GCTAGTAATCGCGGATCAGC consensus
##
## $differences
##   name pairwise.diffs.to.consensus unused.chars
## 1  fwd                          59            0
## 2  rev                          59            0
##
## $distance.matrix
##           fwd       rev
## fwd 0.0000000 0.2335444
## rev 0.2335444 0.0000000
## attr(,"correction")
## [1] "Jukes-Cantor"
##
## $dendrogram
## 'dendrogram' with 2 branches and 2 members total, at height 0.1167722
##
## $indels
## NULL
##
## $stop.codons
```

```
## NULL
##
## $secondary.peak.columns
## NULL
##
## attr(,"class")
## [1] "merged.read"
```

```
merged.reads$consensus
```

```
##   1297-letter "DNAString" instance
## seq: TGACTGCTCTGCCTCCTGATTGAATAATCGTTTG...TGAAGCCGGAATCGCTAGTAATCGCGGATCAG
C
```

```
BrowseSeqs(merged.reads$alignment)
merged.reads$secondary.peak.columns
```

```
## NULL
```

```
merged.reads$differences
```

```
##   name pairwise.diffs.to.consensus unused.chars
## 1  fwd                          59            0
## 2  rev                          59            0
```

```
write.dna(merged.reads,file="Sanger_isolates/138.fasta",format='fasta',nbcol
= -1, colsep = "", colw = 10000000)

fwd = readsangerseq("Sanger_isolates/152F.F.ab1")
rev = readsangerseq("Sanger_isolates/152R.R.ab1")
fwd = primarySeq(fwd)
rev = primarySeq(rev)
rev = reverseComplement(rev)
reads = DNAStringSet(c(as.character(fwd), as.character(rev)))
names(reads) = c('fwd', 'rev')
merged.reads = merge.reads(reads)
```

```
## [1] "Aligning reads"
## [1] "Calling consensus sequence"
## [1] "Calculating differences between reads and consensus"
```

```
names(merged.reads)
```

```
## [1] "consensus"              "alignment"
## [3] "differences"            "distance.matrix"
## [5] "dendrogram"             "indels"
## [7] "stop.codons"            "secondary.peak.columns"
```

```
merged.reads
```

```
## $consensus
##   1288-letter "DNAString" instance
## seq: GCTCACCGGAAAGAGGAGTGGCGAACGGGTGAGT...TGAAGCCGGAATCGCTAGTAATCGCGGATCAG
```

```
C
##
## $alignment
##   A DNAStringSet instance of length 3
##     width seq                                                 names
## [1]  1288 GCTCACCGGAAAGAGGAGTGG...--------------------- fwd
## [2]  1288 ---------------------...GCTAGTAATCGCGGATCAGC rev
## [3]  1288 GCTCACCGGAAAGAGGAGTGG...GCTAGTAATCGCGGATCAGC consensus
##
## $differences
##   name pairwise.diffs.to.consensus unused.chars
## 1  fwd                           8            0
## 2  rev                           8            0
##
## $distance.matrix
##            fwd        rev
## fwd 0.00000000 0.04859999
## rev 0.04859999 0.00000000
## attr(,"correction")
## [1] "Jukes-Cantor"
##
## $dendrogram
## 'dendrogram' with 2 branches and 2 members total, at height 0.0243
##
## $indels
## NULL
##
## $stop.codons
## NULL
##
## $secondary.peak.columns
## NULL
##
## attr(,"class")
## [1] "merged.read"
```

```
merged.reads$consensus
```

```
##   1288-letter "DNAString" instance
## seq: GCTCACCGGAAAGAGGAGTGGCGAACGGGTGAGT...TGAAGCCGGAATCGCTAGTAATCGCGGATCAG
C
```

```
BrowseSeqs(merged.reads$alignment)
merged.reads$secondary.peak.columns
```

```
## NULL
```

```
merged.reads$differences
```

```
##   name pairwise.diffs.to.consensus unused.chars
## 1  fwd                           8            0
## 2  rev                           8            0
```

```
write.dna(merged.reads,file="Sanger_isolates/152.fasta",format='fasta',nbcol
= -1, colsep = "", colw = 10000000)

fwd = readsangerseq("Sanger_isolates/142F.F.ab1")
rev = readsangerseq("Sanger_isolates/142R.R.ab1")
fwd = primarySeq(fwd)
rev = primarySeq(rev)
rev = reverseComplement(rev)
reads = DNAStringSet(c(as.character(fwd), as.character(rev)))
names(reads) = c('fwd', 'rev')
merged.reads = merge.reads(reads)
```

```
## [1] "Aligning reads"
## [1] "Calling consensus sequence"
## [1] "Calculating differences between reads and consensus"
```

```
names(merged.reads)
```

```
## [1] "consensus"              "alignment"
## [3] "differences"            "distance.matrix"
## [5] "dendrogram"             "indels"
## [7] "stop.codons"            "secondary.peak.columns"
```

```
merged.reads
```

```
## $consensus
##   733-letter "DNAString" instance
## seq: AGTCTTCTAGTAGMRGSKTRSTRSWTCGCYSASG...RAWTCGSYRWAATSGTRWWMAGATTGAACGCT
C
##
## $alignment
##   A DNAStringSet instance of length 3
##     width seq                                               names
## [1]   733 -----------AGCAGCTTGC...GGTATAAAGATTGAACGCTC fwd
## [2]   733 AGTCTTCTAGTAGAGGGGTAG...CGTGATCAG----------- rev
## [3]   733 AGTCTTCTAGTAGMRGSKTRS...SGTRWWMAGATTGAACGCTC consensus
##
## $differences
##   name pairwise.diffs.to.consensus unused.chars
## 1  fwd                         367            0
## 2  rev                         367            0
##
## $distance.matrix
##          fwd      rev
## fwd 0.000000 1.011149
## rev 1.011149 0.000000
## attr(,"correction")
```

```
## [1] "Jukes-Cantor"
##
## $dendrogram
## 'dendrogram' with 2 branches and 2 members total, at height 0.5055747
##
## $indels
## NULL
##
## $stop.codons
## NULL
##
## $secondary.peak.columns
## NULL
##
## attr(,"class")
## [1] "merged.read"
```

```
merged.reads$consensus
```

```
##   733-letter "DNAString" instance
## seq: AGTCTTCTAGTAGMRGSKTRSTRSWTCGCYSASG...RAWTCGSYRWAATSGTRWWMAGATTGAACGCT
C
```

```
BrowseSeqs(merged.reads$alignment)
merged.reads$secondary.peak.columns
```

```
## NULL
```

```
merged.reads$differences
```

```
##   name pairwise.diffs.to.consensus unused.chars
## 1  fwd                         367            0
## 2  rev                         367            0
```

```
write.dna(merged.reads,file="Sanger_isolates/142.fasta",format='fasta',nbcol
= -1, colsep = "", colw = 10000000)

fwd = readsangerseq("Sanger_isolates/184F.F.ab1")
rev = readsangerseq("Sanger_isolates/184R.R.ab1")
fwd = primarySeq(fwd)
rev = primarySeq(rev)
rev = reverseComplement(rev)
reads = DNAStringSet(c(as.character(fwd), as.character(rev)))
names(reads) = c('fwd', 'rev')
merged.reads = merge.reads(reads)
```

```
## [1] "Aligning reads"
## [1] "Calling consensus sequence"
## [1] "Calculating differences between reads and consensus"
```

```
names(merged.reads)
```

```
## [1] "consensus"              "alignment"
## [3] "differences"            "distance.matrix"
## [5] "dendrogram"             "indels"
## [7] "stop.codons"            "secondary.peak.columns"
```

```r
merged.reads
```

```
## $consensus
##   1281-letter "DNAString" instance
## seq: GCTTGCACTGAGATGGCGACCGGCGCACGGGTGA...GTGAAGCTGGATTCGCTAGTAATCGCGCATCA
G
##
## $alignment
##   A DNAStringSet instance of length 3
##     width seq                                                names
## [1]  1281 GCTTGCACTGAGATGGCGACC...--------------------- fwd
## [2]  1281 ---------------------...CGCTAGTAATCGCGCATCAG rev
## [3]  1281 GCTTGCACTGAGATGGCGACC...CGCTAGTAATCGCGCATCAG consensus
##
## $differences
##   name pairwise.diffs.to.consensus unused.chars
## 1  fwd                         114            0
## 2  rev                         114            0
##
## $distance.matrix
##           fwd       rev
## fwd 0.0000000 0.1589253
## rev 0.1589253 0.0000000
## attr(,"correction")
## [1] "Jukes-Cantor"
##
## $dendrogram
## 'dendrogram' with 2 branches and 2 members total, at height 0.07946267
##
## $indels
## NULL
##
## $stop.codons
## NULL
##
## $secondary.peak.columns
## NULL
##
## attr(,"class")
## [1] "merged.read"
```

```r
merged.reads$consensus
```

```
##   1281-letter "DNAString" instance
## seq: GCTTGCACTGAGATGGCGACCGGCGCACGGGTGA...GTGAAGCTGGATTCGCTAGTAATCGCGCATCA
G
```

```
BrowseSeqs(merged.reads$alignment)
merged.reads$secondary.peak.columns

## NULL

merged.reads$differences

##   name pairwise.diffs.to.consensus unused.chars
## 1  fwd                         114            0
## 2  rev                         114            0

write.dna(merged.reads,file="Sanger_isolates/184.fasta",format='fasta',nbcol
= -1, colsep = "", colw = 10000000)

fwd = readsangerseq("Sanger_isolates/185F.F.ab1")
rev = readsangerseq("Sanger_isolates/185R.R.ab1")
fwd = primarySeq(fwd)
rev = primarySeq(rev)
rev = reverseComplement(rev)
reads = DNAStringSet(c(as.character(fwd), as.character(rev)))
names(reads) = c('fwd', 'rev')
merged.reads = merge.reads(reads)

## [1] "Aligning reads"
## [1] "Calling consensus sequence"
## [1] "Calculating differences between reads and consensus"

names(merged.reads)

## [1] "consensus"             "alignment"
## [3] "differences"           "distance.matrix"
## [5] "dendrogram"            "indels"
## [7] "stop.codons"           "secondary.peak.columns"

merged.reads

## $consensus
##   1286-letter "DNAString" instance
## seq: GCACTGAGATGGCGACCGGCGCACGGGTGAGTAA...CGTGAAGCTGGATTCGCTAGTAATCGCGCATC
A
##
## $alignment
##   A DNAStringSet instance of length 3
##     width seq                                               names
## [1]  1286 GCACTGAGATGGCGACCGGCG...--------------------- fwd
## [2]  1286 ---------------------...TCGCTAGTAATCGCGCATCA rev
## [3]  1286 GCACTGAGATGGCGACCGGCG...TCGCTAGTAATCGCGCATCA consensus
##
## $differences
##   name pairwise.diffs.to.consensus unused.chars
## 1  fwd                          45            0
## 2  rev                          45            0
```

```
##
## $distance.matrix
##             fwd        rev
## fwd 0.00000000 0.07544043
## rev 0.07544043 0.00000000
## attr(,"correction")
## [1] "Jukes-Cantor"
##
## $dendrogram
## 'dendrogram' with 2 branches and 2 members total, at height 0.03772021
##
## $indels
## NULL
##
## $stop.codons
## NULL
##
## $secondary.peak.columns
## NULL
##
## attr(,"class")
## [1] "merged.read"
```

```
merged.reads$consensus
```

```
##   1286-letter "DNAString" instance
## seq: GCACTGAGATGGCGACCGGCGCACGGGTGAGTAA...CGTGAAGCTGGATTCGCTAGTAATCGCGCATC
A
```

```
BrowseSeqs(merged.reads$alignment)
merged.reads$secondary.peak.columns
```

```
## NULL
```

```
merged.reads$differences
```

```
##   name pairwise.diffs.to.consensus unused.chars
## 1  fwd                          45            0
## 2  rev                          45            0
```

```
write.dna(merged.reads,file="Sanger_isolates/185.fasta",format='fasta',nbcol
= -1, colsep = "", colw = 10000000)

fwd = readsangerseq("Sanger_isolates/186F.F.ab1")
rev = readsangerseq("Sanger_isolates/186R.R.ab1")
fwd = primarySeq(fwd)
rev = primarySeq(rev)
rev = reverseComplement(rev)
reads = DNAStringSet(c(as.character(fwd), as.character(rev)))
names(reads) = c('fwd', 'rev')
merged.reads = merge.reads(reads)
```

```
## [1] "Aligning reads"
## [1] "Calling consensus sequence"
## [1] "Calculating differences between reads and consensus"
```

```r
names(merged.reads)
```

```
## [1] "consensus"              "alignment"
## [3] "differences"            "distance.matrix"
## [5] "dendrogram"             "indels"
## [7] "stop.codons"            "secondary.peak.columns"
```

```r
merged.reads
```

```
## $consensus
##   1270-letter "DNAString" instance
## seq: GCAACTGAGATGGCGACCGGCGCACGGGTGAGTA...CGTGAAGCTGGATTCGCTAGTAATCGCGCATC
A
##
## $alignment
##   A DNAStringSet instance of length 3
##     width seq                                          names
## [1]  1270 GCAACTGAGATGGCGACCGGC...--------------------- fwd
## [2]  1270 ---------------------...TCGCTAGTAATCGCGCATCA rev
## [3]  1270 GCAACTGAGATGGCGACCGGC...TCGCTAGTAATCGCGCATCA consensus
##
## $differences
##   name pairwise.diffs.to.consensus unused.chars
## 1  fwd                          26            0
## 2  rev                          26            0
##
## $distance.matrix
##            fwd        rev
## fwd 0.00000000 0.07729607
## rev 0.07729607 0.00000000
## attr(,"correction")
## [1] "Jukes-Cantor"
##
## $dendrogram
## 'dendrogram' with 2 branches and 2 members total, at height 0.03864803
##
## $indels
## NULL
##
## $stop.codons
## NULL
##
## $secondary.peak.columns
## NULL
##
## attr(,"class")
## [1] "merged.read"
```

```
merged.reads$consensus

##   1270-letter "DNAString" instance
## seq: GCAACTGAGATGGCGACCGGCGCACGGGTGAGTA...CGTGAAGCTGGATTCGCTAGTAATCGCGCATC
A

BrowseSeqs(merged.reads$alignment)
merged.reads$secondary.peak.columns

## NULL

merged.reads$differences

##   name pairwise.diffs.to.consensus unused.chars
## 1  fwd                          26            0
## 2  rev                          26            0

write.dna(merged.reads,file="Sanger_isolates/186.fasta",format='fasta',nbcol
= -1, colsep = "", colw = 10000000)
```

## Most similar OTUs for each Sanger read

A reference alignment was built from the Sanger reads (for each donor separately) applying the sina alinger (Pruesse et al. 2012). OTUs were aligned to this reference Sanger alignment in mothur (align.seqs), yielding a report with the closest Sanger reference for each OTU based on kmer searching (Schloss et al. 2009). The report for each donor was loaded into R, version 3.4.2 (2017-09-28) (R Core Team 2016). For each isolate in the report, the top two OTUs with the highest SearchScores were selected. A fasta file was constructed containing these OTUs.

```
# Donor1
donor1topotusanger <- read.table('DataS13.report',header=TRUE)
donor1topotusanger <- donor1topotusanger[order(donor1topotusanger$TemplateNam
e,-donor1topotusanger$SearchScore),]
donor1topotusanger <-donor1topotusanger%>%group_by(TemplateName) %>%dplyr::sl
ice(1:2)
# find these otu's in the fasta
donor1fastaotusforfylogeny <- read.table(DataS17.fasta')
donor1fastaotusforfylogeny <- donor1fastaotusforfylogeny[donor1fastaotusforfy
logeny$V1%in%donor1topotusanger$QueryName,]
wantedforfylogeny1 <-paste(RDPtax_noprob[donor1fastaotusforfylogeny$V1,]$Genu
s,donor1fastaotusforfylogeny$V1,sep=" ")
donor1fastaotusforfylogeny$V1 <- paste(RDPtax_noprob[donor1fastaotusforfyloge
ny$V1,]$Genus,donor1fastaotusforfylogeny$V1,sep="_")
write.csv2(donor1fastaotusforfylogeny,"donor1fastaotusforfylogeny.csv",row.na
mes=FALSE,col.names=FALSE)

# Donor2
donor2topotusanger <- read.table('DataS14.report',header=TRUE)
donor2topotusanger <- donor2topotusanger[order(donor2topotusanger$TemplateNam
e,-donor2topotusanger$SearchScore),]
```

```
donor2topotusanger <-donor2topotusanger%>%group_by(TemplateName) %>%dplyr::sl
ice(1:2)
donor2fastaotusforfylogeny <- read.table('DataS17.fasta')
donor2fastaotusforfylogeny <- donor2fastaotusforfylogeny[donor2fastaotusforfy
logeny$V1%in%donor2topotusanger$QueryName,]
wantedforfylogeny2 <-paste(RDPtax_noprob[donor2fastaotusforfylogeny$V1,]$Genu
s,donor2fastaotusforfylogeny$V1,sep=" ")
donor2fastaotusforfylogeny$V1 <- paste(RDPtax_noprob[donor2fastaotusforfyloge
ny$V1,]$Genus,donor2fastaotusforfylogeny$V1,sep="_")
write.csv2(donor2fastaotusforfylogeny,"donor2fastaotusforfylogeny.csv",row.na
mes=FALSE,col.names=FALSE)

# Donor3
donor3topotusanger <- read.table('DataS15.report',header=TRUE)
donor3topotusanger <- donor3topotusanger[order(donor3topotusanger$TemplateNam
e,-donor3topotusanger$SearchScore),]
donor3topotusanger <-donor3topotusanger%>%group_by(TemplateName) %>%dplyr::sl
ice(1:2)
donor3fastaotusforfylogeny <- read.table('DataS17.fasta')
donor3fastaotusforfylogeny <- donor3fastaotusforfylogeny[donor3fastaotusforfy
logeny$V1%in%donor3topotusanger$QueryName,]
wantedforfylogeny3 <-paste(RDPtax_noprob[donor3fastaotusforfylogeny$V1,]$Genu
s,donor3fastaotusforfylogeny$V1,sep=" ")
donor3fastaotusforfylogeny$V1 <- paste(RDPtax_noprob[donor3fastaotusforfyloge
ny$V1,]$Genus,donor3fastaotusforfylogeny$V1,sep="_")
write.csv2(donor3fastaotusforfylogeny,"donor3fastaotusforfylogeny.csv",row.na
mes=FALSE,col.names=FALSE)


# Donor4
donor4topotusanger <- read.table('DataS16.report',header=TRUE)
donor4topotusanger <- donor4topotusanger[order(donor4topotusanger$TemplateNam
e,-donor4topotusanger$SearchScore),]
donor4topotusanger <-donor4topotusanger%>%group_by(TemplateName) %>%dplyr::sl
ice(1:2)
donor4fastaotusforfylogeny <- read.table('DataS18.fasta')
donor4fastaotusforfylogeny <- donor4fastaotusforfylogeny[donor4fastaotusforfy
logeny$V1%in%donor4topotusanger$QueryName,]
wantedforfylogeny4 <-paste(RDPtax4_noprob[donor4fastaotusforfylogeny$V1,]$Gen
us,donor4fastaotusforfylogeny$V1,sep=" ")
donor4fastaotusforfylogeny$V1 <- paste(RDPtax4_noprob[donor4fastaotusforfylog
eny$V1,]$Genus,donor4fastaotusforfylogeny$V1,sep="_")
write.csv2(donor4fastaotusforfylogeny,"donor4fastaotusforfylogeny.csv",row.na
mes=FALSE,col.names=FALSE)
```

## Create metadata for the annotation of ITOL phylogenetic trees

In order to compare the OTUs spanning the V4 region of the 16S rRNA gene with the near full-length Sanger reads, the RAxML implementation of the evolutionary placement algorithm of short reads, as introduced by Berger et al. (2011), was used (Stamatakis 2014). The bootstrap supported maximum likelihood (ML) phylogenetic reference tree was also constructed using RAxML, selecting the General Time Reversible model of nucleotide substitution under the Gamma model of rate heterogeneity (GTRGAMMA) with the parsimony random seed set to 12345. The rapid bootstrap analysis was conducted starting from N=1000 distinct randomized maximum parsimony trees and was followed by a search for the best-scoring ML tree with rapid bootstrap random number seed 123 (Stamatakis 2014). The best scoring ML tree with the OTU short read insertions was visualized in iTOL (Letunic & Bork 2016). Metadata for the annotation of the iTOL trees is created in the chuncks below.

```
currentotuname1 <- donor1fastaotusforfylogeny$V1
RAXMLotuname1 <- paste("QUERY___",currentotuname1,sep="")
otuname1df <- cbind(currentname1=RAXMLotuname1,wantedname1=wantedforfylogeny1
)

wantedsangername1 <- splittax(read.table('DataS19.taxonomy'),"seqs")
wantedsangername1$Genus <- mapvalues(wantedsangername1$Genus,from=c("Enteroba
cteriaceae_unclassified","Erysipelotrichaceae_unclassified","Lachnospiraceae_
unclassified","Lactobacillales_unclassified","Actinobacteria_unclassified","B
urkholderiales_unclassified","Clostridiales_unclassified"),to=c("Enterobacter
iaceae","Erysipelotrichaceae","Lachnospiraceae","Lactobacillales","Actinobact
eria","Burkholderiales","Clostridiales"))
currentsangername1 <-as.character(wantedsangername1$id)
wantedsangername1 <- as.character(paste(wantedsangername1$id,wantedsangername
1$Genus))
sangername1df <- cbind(currentname1=currentsangername1,wantedname1=wantedsang
ername1)

itolname1 <- data.frame(rbind(otuname1df,sangername1df))
itolname1$itolcode <- paste(itolname1$currentname1,",",itolname1$wantedname1,
sep="")
itollabel1<- c("LABELS","SEPARATOR COMMA","DATA",itolname1$itolcode)
write.table(itollabel1,"itollabel1.txt",row.names=FALSE,col.names=FALSE,quote
=FALSE)

#style labels
itollabelstyle1 <- paste(currentsangername1,",label",",#000000",",bold",",1",
sep="")
itollabelstyle1<- c("TREE_COLORS","SEPARATOR COMMA","DATA",itollabelstyle1)
write.table(itollabelstyle1,"itollabelstyle1.txt",row.names=FALSE,col.names=F
ALSE,quote=FALSE)

# Stacked bar charts of the relative abundance
propbar1 <- subset(shared3minsingletonwnwnprop*100,select=c(Donor1_high4_L.V4
```

```
,Donor1_low4_L.V4,FS_1_isolationhigh.V4))
propbar1 <- propbar1[rownames(propbar1)%in%donor1topotusanger$QueryName,]
propbar1 <- round(propbar1,2)
propbar1 <- rownames_to_column(propbar1)
propbar1$rowname <-paste(RDPtax_noprob[propbar1$rowname,]$Genus,propbar1$rown
ame,sep="_")
propbar1$rowname<- paste("QUERY___",propbar1$rowname,sep="")
propbar1$high <- paste(",",propbar1$Donor1_high4_L.V4,sep="")
propbar1$low<- paste(",",propbar1$Donor1_low4_L.V4,sep="")
propbar1$FS <- paste(",",propbar1$FS_1_isolationhigh.V4,sep="")
propbar1 <- propbar1[,-c(2:4)]
propbar1 <- rbind(c("DATA","","",""),propbar1)
propbar1 <- rbind(c("DATASET_SCALE",",10",",25",",50"),propbar1)
propbar1 <- rbind(c("ALIGN_FIELDS",",0","",""),propbar1)
propbar1 <- rbind(c("LEGEND_TITLE",",Relative abundance (%)","",""),propbar1)
propbar1 <- rbind(c("LEGEND_SHAPES",",1",",1",",1"),propbar1)
propbar1 <- rbind(c("LEGEND_COLORS",",#323C4D",",#A03D44",",#98B736"),propbar
1)
propbar1 <- rbind(c("LEGEND_LABELS",",pH6.8",",pH5.8",",Fecal slurry"),propba
r1)
propbar1 <- rbind(c("WIDTH",",350","",""),propbar1)
propbar1 <- rbind(c("FIELD_COLORS",",#323C4D",",#A03D44",",#98B736"),propbar1
)
propbar1 <- rbind(c("FIELD_LABELS",",f1",",f2",",f3"),propbar1)
propbar1 <- rbind(c("COLOR",",#ff0000","",""),propbar1)
propbar1 <- rbind(c("DATASET_LABEL",",barchart","",""),propbar1)
propbar1 <- rbind(c("SEPARATOR"," COMMA","",""),propbar1)
propbar1 <- rbind(c("DATASET_MULTIBAR","","",""),propbar1)
write.table(propbar1,"itolpropbar1.txt",col.names=FALSE,row.names=FALSE,sep="
",quote=FALSE)

currentotuname2 <- donor2fastaotusforfylogeny$V1
RAXMLotuname2 <- paste("QUERY___",currentotuname2,sep="")
otuname2df <- cbind(currentname2=RAXMLotuname2,wantedname2=wantedforfylogeny2
)

wantedsangername2 <- splittax(read.table('DataS20.taxonomy'),"seqs")
wantedsangername2$Genus <- mapvalues(wantedsangername2$Genus,from=c("Enteroba
cteriaceae_unclassified","Erysipelotrichaceae_unclassified","Lachnospiraceae_
unclassified","Lactobacillales_unclassified","Actinobacteria_unclassified","B
urkholderiales_unclassified","Clostridiales_unclassified"),to=c("Enterobacter
iaceae","Erysipelotrichaceae","Lachnospiraceae","Lactobacillales","Actinobact
eria","Burkholderiales","Clostridiales"))
currentsangername2 <-as.character(wantedsangername2$id)
wantedsangername2 <- as.character(paste(wantedsangername2$id,wantedsangername
2$Genus))
sangername2df <- cbind(currentname2=currentsangername2,wantedname2=wantedsang
ername2)

itolname2 <- data.frame(rbind(otuname2df,sangername2df))
```

```
itolname2$itolcode <- paste(itolname2$currentname2,",",itolname2$wantedname2,
sep="")
itollabel2<- c("LABELS","SEPARATOR COMMA","DATA",itolname2$itolcode)
write.table(itollabel2,"itollabel2.txt",row.names=FALSE,col.names=FALSE,quote
=FALSE)

#style labels
itollabelstyle2 <- paste(currentsangername2,",label",",#000000",",bold",",1",
sep="")
itollabelstyle2<- c("TREE_COLORS","SEPARATOR COMMA","DATA",itollabelstyle2)
write.table(itollabelstyle2,"itollabelstyle2.txt",row.names=FALSE,col.names=F
ALSE,quote=FALSE)

# Stacked bar charts of the relative abundance
propbar2 <- subset(shared3minsingletonwnwnprop*100,select=c(Donor2_high4_L.V4
,Donor2_low4_L.V4,FS_2_isolationhigh.V4))
propbar2 <- propbar2[rownames(propbar2)%in%donor2topotusanger$QueryName,]
propbar2 <- round(propbar2,2)
propbar2 <- rownames_to_column(propbar2)
propbar2$rowname <-paste(RDPtax_noprob[propbar2$rowname,]$Genus,propbar2$rown
ame,sep="_")
propbar2$rowname<- paste("QUERY___",propbar2$rowname,sep="")
propbar2$high <- paste(",",propbar2$Donor2_high4_L.V4,sep="")
propbar2$low<- paste(",",propbar2$Donor2_low4_L.V4,sep="")
propbar2$FS <- paste(",",propbar2$FS_2_isolationhigh.V4,sep="")
propbar2 <- propbar2[,-c(2:4)]
propbar2 <- rbind(c("DATA","","",""),propbar2)
propbar2 <- rbind(c("DATASET_SCALE",",5",",10",",25"),propbar2)
propbar2 <- rbind(c("ALIGN_FIELDS",",0","",""),propbar2)
propbar2 <- rbind(c("LEGEND_TITLE",",Relative abundance (%)","",""),propbar2)
propbar2 <- rbind(c("LEGEND_SHAPES",",1",",1",",1"),propbar2)
propbar2 <- rbind(c("LEGEND_COLORS",",#323C4D",",#A03D44",",#98B736"),propbar
2)
propbar2 <- rbind(c("LEGEND_LABELS",",pH6.8",",pH5.8",",Fecal slurry"),propba
r2)
propbar2 <- rbind(c("WIDTH",",350","",""),propbar2)
propbar2 <- rbind(c("FIELD_COLORS",",#323C4D",",#A03D44",",#98B736"),propbar2
)
propbar2 <- rbind(c("FIELD_LABELS",",f1",",f2",",f3"),propbar2)
propbar2 <- rbind(c("COLOR",",#ff0000","",""),propbar2)
propbar2 <- rbind(c("DATASET_LABEL",",barchart","",""),propbar2)
propbar2 <- rbind(c("SEPARATOR"," COMMA","",""),propbar2)
propbar2 <- rbind(c("DATASET_MULTIBAR","","",""),propbar2)
write.table(propbar2,"itolpropbar2.txt",col.names=FALSE,row.names=FALSE,sep="
",quote=FALSE)

currentotuname3 <- donor3fastaotusforfylogeny$V1
RAXMLotuname3 <- paste("QUERY___",currentotuname3,sep="")
otuname3df <- cbind(currentname3=RAXMLotuname3,wantedname3=wantedforfylogeny3
)
```

```
wantedsangername3 <- splittax(read.table('DataS21.taxonomy'),"seqs")
wantedsangername3$Genus <- mapvalues(wantedsangername3$Genus,from=c("Enterobacteriaceae_unclassified","Erysipelotrichaceae_unclassified","Lachnospiraceae_unclassified","Lactobacillales_unclassified","Actinobacteria_unclassified","Burkholderiales_unclassified","Clostridiales_unclassified"),to=c("Enterobacteriaceae","Erysipelotrichaceae","Lachnospiraceae","Lactobacillales","Actinobacteria","Burkholderiales","Clostridiales"))
currentsangername3 <-as.character(wantedsangername3$id)
wantedsangername3 <- as.character(paste(wantedsangername3$id,wantedsangername3$Genus))
sangername3df <- cbind(currentname3=currentsangername3,wantedname3=wantedsangername3)

itolname3 <- data.frame(rbind(otuname3df,sangername3df))
itolname3$itolcode <- paste(itolname3$currentname3,",",itolname3$wantedname3,sep="")
itollabel3<- c("LABELS","SEPARATOR COMMA","DATA",itolname3$itolcode)
write.table(itollabel3,"itollabel3.txt",row.names=FALSE,col.names=FALSE,quote=FALSE)

#style labels
itollabelstyle3 <- paste(currentsangername3,",label",",#000000",",bold",",1",sep="")
itollabelstyle3<- c("TREE_COLORS","SEPARATOR COMMA","DATA",itollabelstyle3)
write.table(itollabelstyle3,"itollabelstyle3.txt",row.names=FALSE,col.names=FALSE,quote=FALSE)

# Stacked bar charts of the relative abundance
propbar3 <- subset(shared3minsingletonwnwnprop*100,select=c(Donor3_high4_L.V4,Donor3_low4_L.V4,FS_3_isolationhigh.V4))
propbar3 <- propbar3[rownames(propbar3)%in%donor3topotusanger$QueryName,]
propbar3 <- round(propbar3,2)
propbar3 <- rownames_to_column(propbar3)
propbar3$rowname <-paste(RDPtax_noprob[propbar3$rowname,]$Genus,propbar3$rowname,sep="_")
propbar3$rowname<- paste("QUERY___",propbar3$rowname,sep="")
propbar3$high <- paste(",",propbar3$Donor3_high4_L.V4,sep="")
propbar3$low<- paste(",",propbar3$Donor3_low4_L.V4,sep="")
propbar3$FS <- paste(",",propbar3$FS_3_isolationhigh.V4,sep="")
propbar3 <- propbar3[,-c(2:4)]
propbar3 <- rbind(c("DATA","","",""),propbar3)
propbar3 <- rbind(c("DATASET_SCALE",",25",",50",",100"),propbar3)
propbar3 <- rbind(c("ALIGN_FIELDS",",0","",""),propbar3)
propbar3 <- rbind(c("LEGEND_TITLE",",Relative abundance (%)","",""),propbar3)
propbar3 <- rbind(c("LEGEND_SHAPES",",1",",1",",1"),propbar3)
propbar3 <- rbind(c("LEGEND_COLORS",",#323C4D",",#A03D44",",#98B736"),propbar3)
propbar3 <- rbind(c("LEGEND_LABELS",",pH6.8",",pH5.8",",Fecal slurry"),propbar3)
```

```
propbar3 <- rbind(c("WIDTH",",350","",""),propbar3)
propbar3 <- rbind(c("FIELD_COLORS",",#323C4D",",#A03D44",",#98B736"),propbar3
)
propbar3 <- rbind(c("FIELD_LABELS",",f1",",f2",",f3"),propbar3)
propbar3 <- rbind(c("COLOR",",#ff0000","",""),propbar3)
propbar3 <- rbind(c("DATASET_LABEL",",barchart","",""),propbar3)
propbar3 <- rbind(c("SEPARATOR"," COMMA","",""),propbar3)
propbar3 <- rbind(c("DATASET_MULTIBAR","","",""),propbar3)
write.table(propbar3,"itolpropbar3.txt",col.names=FALSE,row.names=FALSE,sep="
",quote=FALSE)

currentotuname4 <- donor4fastaotusforfylogeny$V1
RAXMLotuname4 <- paste("QUERY___",currentotuname4,sep="")
otuname4df <- cbind(currentname4=RAXMLotuname4,wantedname4=wantedforfylogeny4
)

wantedsangername4 <- splittax(read.table('DataS22.taxonomy'),"seqs")
wantedsangername4$Genus <- mapvalues(wantedsangername4$Genus,from=c("Enteroba
cteriaceae_unclassified","Erysipelotrichaceae_unclassified","Lachnospiraceae_
unclassified","Lactobacillales_unclassified","Actinobacteria_unclassified","B
urkholderiales_unclassified","Clostridiales_unclassified","Ruminococcaceae_un
classified","Coriobacteriaceae_unclassified","Lactobacillaceae_unclassified",
"Microbacteriaceae_unclassified"),to=c("Enterobacteriaceae","Erysipelotrichac
eae","Lachnospiraceae","Lactobacillales","Actinobacteria","Burkholderiales","
Clostridiales","Ruminococcaceae","Coriobacteriaceae","Lactobacillaceae","Micr
obacteriaceae"))
currentsangername4 <-as.character(wantedsangername4$id)
wantedsangername4 <- as.character(paste(wantedsangername4$id,wantedsangername
4$Genus))
sangername4df <- cbind(currentname4=currentsangername4,wantedname4=wantedsang
ername4)

itolname4 <- data.frame(rbind(otuname4df,sangername4df))
itolname4$itolcode <- paste(itolname4$currentname4,",",itolname4$wantedname4,
sep="")
itollabel4<- c("LABELS","SEPARATOR COMMA","DATA",itolname4$itolcode)
write.table(itollabel4,"itollabel4.txt",row.names=FALSE,col.names=FALSE,quote
=FALSE)

#style labels
itollabelstyle4 <- paste(currentsangername4,",label",",#000000",",bold",",1",
sep="")
itollabelstyle4<- c("TREE_COLORS","SEPARATOR COMMA","DATA",itollabelstyle4)
write.table(itollabelstyle4,"itollabelstyle4.txt",row.names=FALSE,col.names=F
ALSE,quote=FALSE)

# Stacked bar charts of the relative abundance
propbar4 <- subset(shared4minsingletonwnwnprop*100,select=c(Kim8_Donor4_high4
_L,Kim4_Donor4_low4_L,Kim1_Donor4_FS_high))
propbar4 <- propbar4[rownames(propbar4)%in%donor4topotusanger$QueryName,]
```

```
propbar4 <- round(propbar4,2)
propbar4 <- rownames_to_column(propbar4)
propbar4$rowname <-paste(RDPtax4_noprob[propbar4$rowname,]$Genus,propbar4$rowname,sep="_")
propbar4$rowname<- paste("QUERY___",propbar4$rowname,sep="")
propbar4$high <- paste(",",propbar4$Kim8_Donor4_high4_L,sep="")
propbar4$low<- paste(",",propbar4$Kim4_Donor4_low4_L,sep="")
propbar4$FS <- paste(",",propbar4$Kim1_Donor4_FS_high,sep="")
propbar4 <- propbar4[,-c(2:4)]
propbar4 <- rbind(c("DATA","","",""),propbar4)
propbar4 <- rbind(c("DATASET_SCALE",",25",",50",",100"),propbar4)
propbar4 <- rbind(c("ALIGN_FIELDS",",0","",""),propbar4)
propbar4 <- rbind(c("LEGEND_TITLE",",Relative abundance (%)","",""),propbar4)
propbar4 <- rbind(c("LEGEND_SHAPES",",1",",1",",1"),propbar4)
propbar4 <- rbind(c("LEGEND_COLORS",",#323C4D",",#A03D44",",#98B736"),propbar4)
propbar4 <- rbind(c("LEGEND_LABELS",",pH6.8",",pH5.8",",Fecal slurry"),propbar4)
propbar4 <- rbind(c("WIDTH",",350","",""),propbar4)
propbar4 <- rbind(c("FIELD_COLORS",",#323C4D",",#A03D44",",#98B736"),propbar4)
propbar4 <- rbind(c("FIELD_LABELS",",f1",",f2",",f3"),propbar4)
propbar4 <- rbind(c("COLOR",",#ff0000","",""),propbar4)
propbar4 <- rbind(c("DATASET_LABEL",",barchart","",""),propbar4)
propbar4 <- rbind(c("SEPARATOR"," COMMA","",""),propbar4)
propbar4 <- rbind(c("DATASET_MULTIBAR","","",""),propbar4)
write.table(propbar4,"itolpropbar4.txt",col.names=FALSE,row.names=FALSE,sep="
",quote=FALSE)
```
