# Supplementary material for: Isolation of wheat bran-colonizing and metabolizing species from the human fecal microbiota
Source: PeerJ. 2019 Jan 25;7:e6293. doi: 10.7717/peerj.6293 (PMC6348960; doi:10.7717/peerj.6293)
Supplement: Supplemental Information 1 [file peerj-07-6293-s023.docx]

**Supplementary materials and methods: wheat bran characterization**

The average particle size of bran was measured by sieving 20 g of dry bran on a set of sieves with different mesh sizes and weighing the amount of bran remaining on each sieve (Jacobs et al. 2015). The water retention capacity was determined in triplicate according to AACC (American Association of Cereal Chemists) international method 56-11-02 (AACC 1999). The strongly bound water was estimated using a drainage centrifugation technique Jacobs et al. (2015). The arabinoxylan, starch, β-glucan, fructan, cellulose, lipid, protein and ash levels were measured in triplicate and the DF levels were measured in duplicate as described by De Paepe et al. (2017). Briefly, the dietary fibre level of wheat bran was determined according to AOAC Method 991.43 using the Megazyme dietary fiber assay (K-TDFR, Megazyme, Bray, Ireland) (AOAC 1995). β-glucan was determined with the Megazyme mixed linkage β-glucan kit (K-BGLU, Megazyme, Bray, Ireland), as described in AACC method 32-23 (AACC 1999). Fructan was quantified by high performance anion exchange chromatography with pulsed amperometric detection (HPAEC-PAD) on a Dionex ICS3000 chromatography system (Sunnyvale, CA, USA) after mild acid hydrolysis (Verspreet et al. 2012). The remaining sugars were measured after acid hydrolysis using sulfuric acid (cellulose) and trifluoroacetic acid (arabinoxylans and starch), followed by sugar derivatization and gas chromatographic analysis. Protein concentrations were measured by the Dumas combustion method, an adapted version of the AOAC Official Method 990.03 (AOAC 1995). An automated Dumas protein analysis system (EAS VarioMax N/CN, Elt, Gouda, The Netherlands) was used with 6.25 as the nitrogen to protein conversion factor. Lipid content was gravimetrically determined after extraction of lipids from bran with water saturated butanol using an ASE 200 device (Dionex, Amsterdam, The Netherlands) and a subsequent purification step (Gerits et al. 2013). Ash content was measured with AACC method 08-01.01 (AACC 1999)”.

Prior to in vitro fermentation, all wheat bran products underwent in vitro batch digestion, according to an adaptation to the method from Minekus et al. (2014), described by De Paepe et al. (2017), to mimic stomach and small intestine passage. Briefly, wheat bran samples were incubated at 37°C with simulated saliva fluid containing salivary α-amylase at pH 7 for 15 min (1:2 w/v), simulated gastric fluid containing porcine pepsin at pH 3 for 2 h (1:1 v/v) and simulated pancreatic juice containing porcine pancreatin and bile salts at pH 7 for 2 h (1:1 w/v). Insoluble, undigested material was collected by centrifugation at 500g for 5 min and lyophilised for preservation until chemical characterisation or batch incubation with faecal slurries from the ten donors.

AACC. 1999. *Approved Methods of the American Association of Cereal Chemists*. AACC, Inc., St. Paul, MN, USA.

AOAC. 1995. *Official Methods of Analysis of the Association of Official Analytical Chemists*. The Association of Official Analytical Chemists, Washington, DC, USA.

De Paepe K, Kerckhof F-M, Verspreet J, Courtin CM, and Van de Wiele T. 2017. Inter-individual differences determine the outcome of wheat bran colonization by the human gut microbiome. *Environmental Microbiology* 19:3251-3267. 10.1111/1462-2920.13819

Gerits LR, Pareyt B, and Delcour JA. 2013. Single run HPLC separation coupled to evaporative light scattering detection unravels wheat flour endogenous lipid redistribution during bread dough making. *Lwt-Food Science and Technology* 53:426-433. 10.1016/j.lwt.2013.03.015

Jacobs PJ, Hemdane S, Dornez E, Delcour JA, and Courtin CM. 2015. Study of hydration properties of wheat bran as a function of particle size. *Food Chemistry* 179:296-304. 10.1016/j.foodchem.2015.01.117

Minekus M, Alminger M, Alvito P, Ballance S, Bohn T, Bourlieu C, Carriere F, Boutrou R, Corredig M, Dupont D, Dufour C, Egger L, Golding M, Karakaya S, Kirkhus B, Le Feunteun S, Lesmes U, Macierzanka A, Mackie A, Marze S, McClements DJ, Menard O, Recio I, Santos CN, Singh RP, Vegarud GE, Wickham MS, Weitschies W, and Brodkorb A. 2014. A standardised static *in vitro* digestion method suitable for food - an international consensus. *Food Funct* 5:1113-1124. 10.1039/c3fo60702j

Verspreet J, Pollet A, Cuyvers S, Vergauwen R, Van den Ende W, Delcour JA, and Courtin CM. 2012. A Simple and Accurate Method for Determining Wheat Grain Fructan Content and Average Degree of Polymerization. *Journal of Agricultural and Food Chemistry* 60:2102-2107. 10.1021/jf204774n
