# Supplementary material for: Isolation of wheat bran-colonizing and metabolizing species from the human fecal microbiota
Source: PeerJ. 2019 Jan 25;7:e6293. doi: 10.7717/peerj.6293 (PMC6348960; doi:10.7717/peerj.6293)
Supplement: Table S1 — 1 mL of the stock solution is added to 1 L medium prior to autoclaving. [file peerj-07-6293-s024.docx]

| **Compound** | **Amount (g L^-1^)** |
| --- | --- |
| Biotin (vit H/vit B8) | 0.01 |
| Cobalamin (vit B12) | 0.01 |
| p-aminobenzoic acid | 0.03 |
| Folic acid (vit B9) | 0.05 |
| Pyridoxamine (vit B6) | 0.15 |
