# Supplementary material for: Isolation of wheat bran-colonizing and metabolizing species from the human fecal microbiota
Source: PeerJ. 2019 Jan 25;7:e6293. doi: 10.7717/peerj.6293 (PMC6348960; doi:10.7717/peerj.6293)
Supplement: Table S2 — 1 mL of the filter sterilized solution (0.22 µm sterile syringe filter, Merck Millipore, Burlington, MA, US) is added to 1 L medium after autoclaving, right before use. [file peerj-07-6293-s025.docx]

| **Compound** | **Amount (g L^-1^)** |
| --- | --- |
| Thiamin (vit B1) | 0.05 |
| Riboflavin (vit B2) | 0.05 |
