# Supplementary material for: Isolation of wheat bran-colonizing and metabolizing species from the human fecal microbiota
Source: PeerJ. 2019 Jan 25;7:e6293. doi: 10.7717/peerj.6293 (PMC6348960; doi:10.7717/peerj.6293)
Supplement: Table S3 — 15 mL of this solution was freshly prepared for each use by adding filter sterilized demineralized water (0.22 µm sterile syringe filter, Merck Millipore, Burlington, MA, US ) to the weighed compounds in the anaerobic workstation. [file peerj-07-6293-s026.docx]

|  | **pH 6.8** | **pH 5.8** |
| --- | --- | --- |
| **Compound** | **Amount (g/15 mL)** | **Amount (g/15 mL)** |
| NaHCO_3_ | 0.4 | 0.25 |
| Cysteine-HCl | 0.3 | 0.3 |
