# Supplementary material for: Isolation of wheat bran-colonizing and metabolizing species from the human fecal microbiota
Source: PeerJ. 2019 Jan 25;7:e6293. doi: 10.7717/peerj.6293 (PMC6348960; doi:10.7717/peerj.6293)
Supplement: Table S4 — 1 mL sample is mixed with 1 mL of the cryoprotective agent. [file peerj-07-6293-s027.docx]

| **Product** | **Amount** |
| --- | --- |
| Glycerol (84%) | 500 mL |
| Cysteine-HCl | 1 g |
| D-(+)-Trehalose-dihydrate  (from *Saccharomyces cerevisiae*, ≥ 99%) | 20 g |
| Tryptic Soy broth | 6 g |
| H_2_O | 500 mL |
