# Supplementary figures and images for: Isolation of wheat bran-colonizing and metabolizing species from the human fecal microbiota
Source: PeerJ. 2019 Jan 25;7:e6293. doi: 10.7717/peerj.6293 (PMC6348960; doi:10.7717/peerj.6293)

## Relative abundance (%)

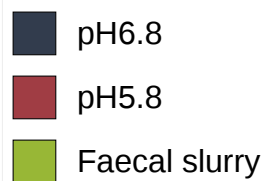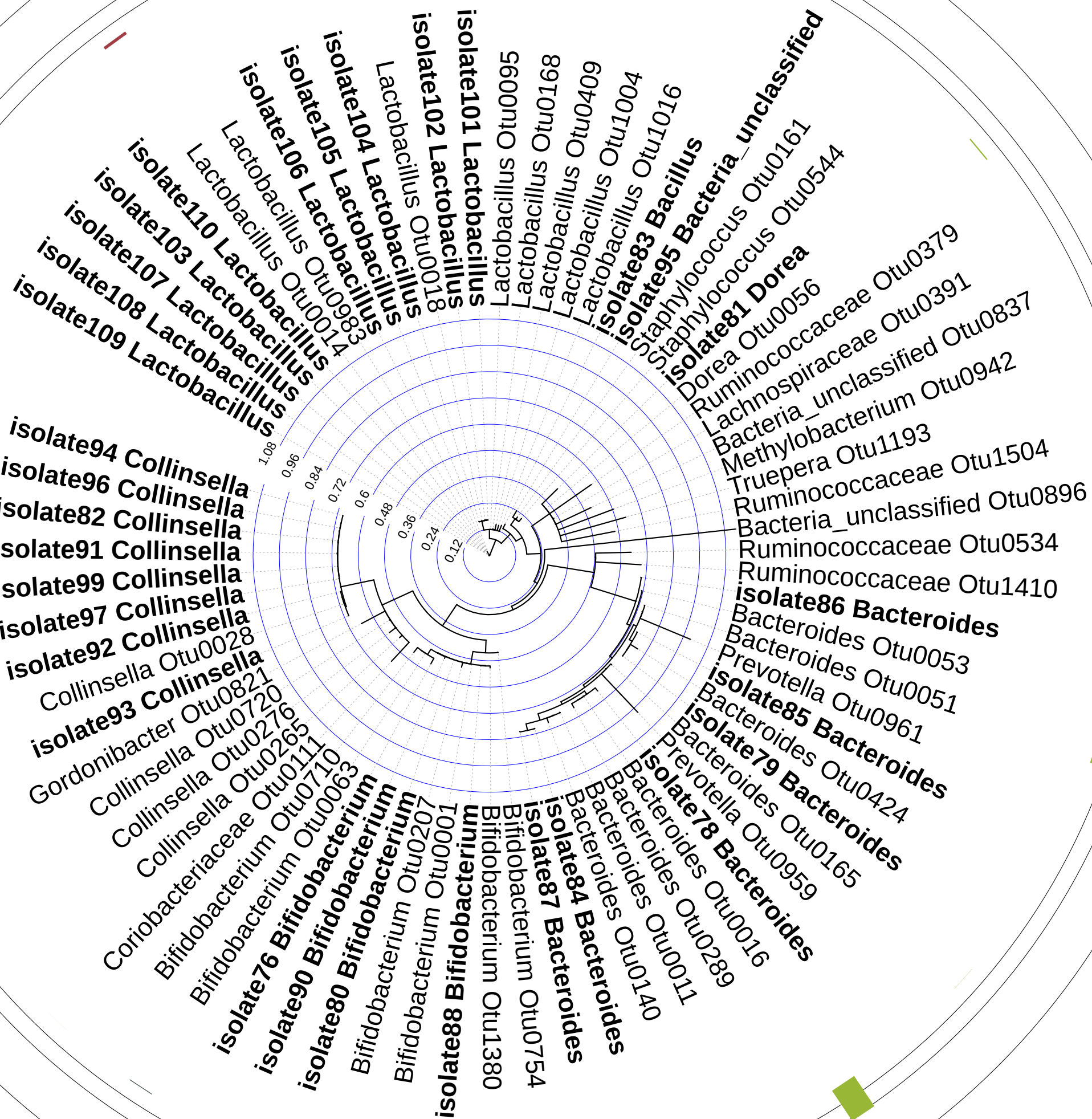

Supplement: Figure S1 [file peerj-07-6293-s028.pdf]

Tree scale: 0.1

Relative abundance (%)

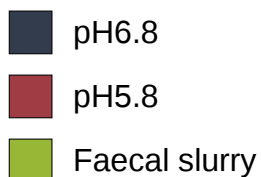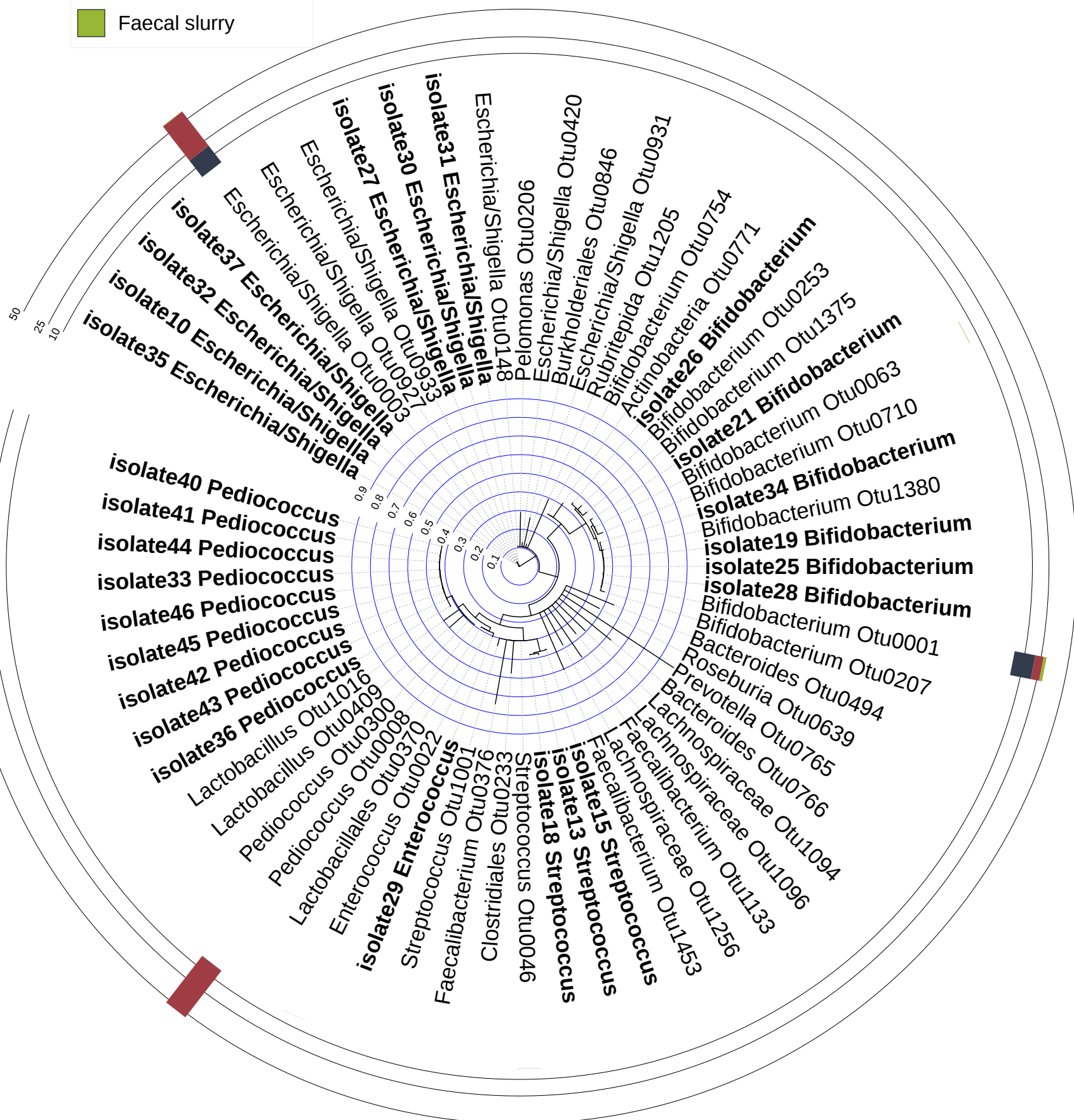

Supplement: Figure S2 [file peerj-07-6293-s029.pdf]

---

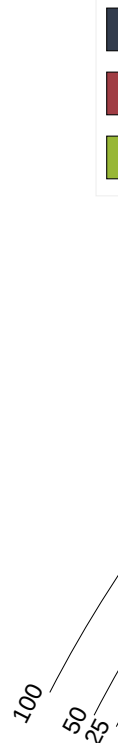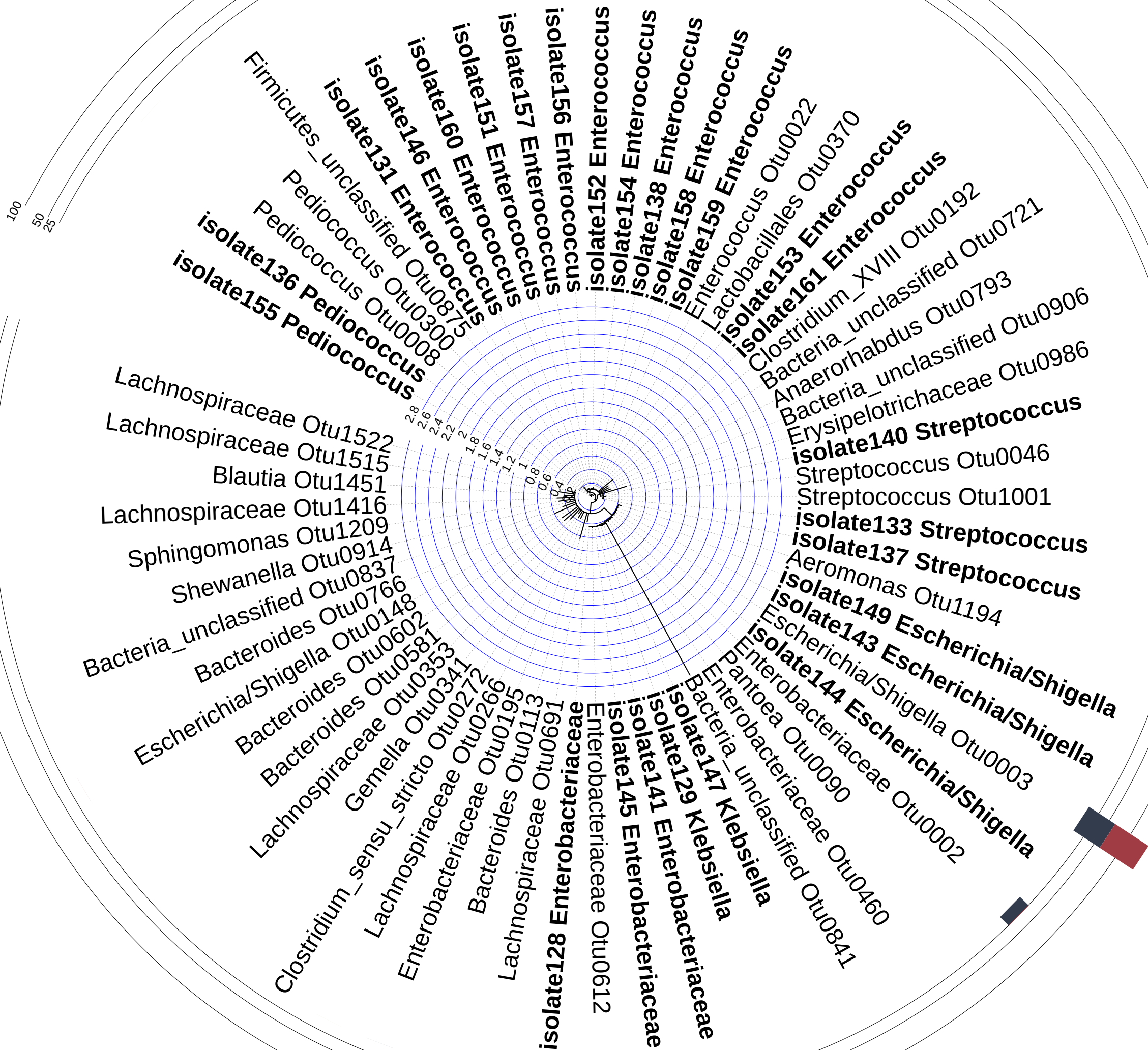

Supplement: Figure S3 [file peerj-07-6293-s030.pdf]
