# Supplementary material for: Isolation of wheat bran-colonizing and metabolizing species from the human fecal microbiota
Source: PeerJ. 2019 Jan 25;7:e6293. doi: 10.7717/peerj.6293 (PMC6348960; doi:10.7717/peerj.6293)
Supplement: Figure S4 [file peerj-07-6293-s031.pdf]

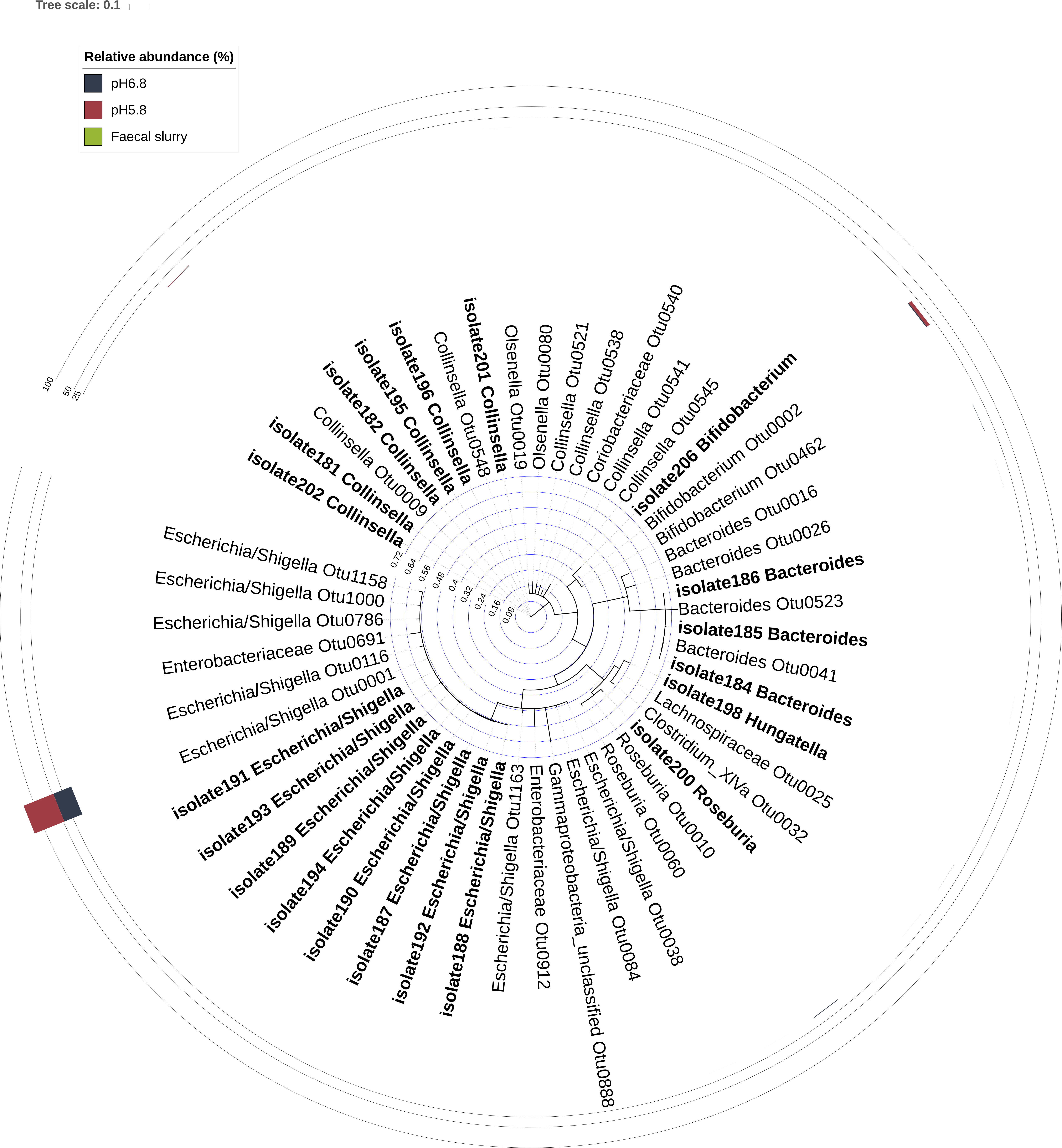

Tree scale: 0.1
Relative abundance (%)
pH6.8
pH5.8
Faecal slurry
100
50
25
0.72
0.64
0.56
0.48
0.4
0.32
0.24
0.16
0.08
isolate202 Collinsella
isolate181 Collinsella
Collinsella Otu0009
isolate182 Collinsella
isolate195 Collinsella
isolate196 Collinsella
Collinsella Otu0548
isolate201 Collinsella
Olsenella Otu0019
Olsenella Otu0080
Collinsella Otu0521
Collinsella Otu0538
Coriobacteriaceae Otu0540
Collinsella Otu0541
Collinsella Otu0545
isolate206 Bifidobacterium
Bifidobacterium Otu0002
Bifidobacterium Otu0462
Bacteroides Otu0016
Bacteroides Otu0026
isolate186 Bacteroides
Bacteroides Otu0523
isolate185 Bacteroides
Bacteroides Otu0041
isolate184 Bacteroides
isolate198 Hungatella
Lachnospiraceae Otu0025
Clostridium_XlVa Otu0032
isolate200 Roseburia
Roseburia Otu0010
Roseburia Otu0060
Escherichia/Shigella Otu0038
Escherichia/Shigella Otu0084
Gammaproteobacteria_unclassified Otu0888
Enterobacteriaceae Otu0912
Escherichia/Shigella Otu1163
isolate188 Escherichia/Shigella
isolate192 Escherichia/Shigella
isolate187 Escherichia/Shigella
isolate190 Escherichia/Shigella
isolate194 Escherichia/Shigella
isolate189 Escherichia/Shigella
isolate193 Escherichia/Shigella
isolate191 Escherichia/Shigella
Escherichia/Shigella Otu0001
Escherichia/Shigella Otu0116
Enterobacteriaceae Otu0691
Escherichia/Shigella Otu0786
Escherichia/Shigella Otu1000
Escherichia/Shigella Otu1158
